# Theoretical investigation of a potentially important formation pathway of organosulfate in atmospheric aqueous aerosols

**DOI:** 10.1038/s41598-020-61968-2

**Published:** 2020-04-14

**Authors:** Kunpeng Chen, Jun Zhao

**Affiliations:** 1 School of Atmospheric Sciences, Sun Yat-sen University, Guangzhou, Guangdong 510275 China; 2 Guangdong Province Key Laboratory for Climate Change and Natural Disaster Studies, and Institute of Earth Climate and Environment System, Sun Yat-sen University, Guangzhou, Guangdong 510275 China; 3 Southern Laboratory of Ocean Science and Engineering (Guangdong, Zhuhai), Zhuhai, Guangdong 519082 China; 4 Present Address: Department of Environmental Sciences, College of Natural and Agricultural Sciences, University of California Riverside, Riverside, CA 92521 USA

**Keywords:** Environmental chemistry, Environmental impact

## Abstract

Organic sulfate plays important roles in modulating properties of atmospheric aerosols. Recent studies showed that organic sulfate was likably interpreted as inorganic sulfate in field measurements using advanced instruments such as Aerosol Mass Spectrometer and the major contributor to organic sulfate was thought to be hydroxymethanesulfonate (HMS). This study proposed that besides HMS, its isomer hydroxymethyl sulfite (HMSi), which has not been identified in atmospheric aerosols, can emerge as the product of aqueous reactions between sulfur dioxide and formaldehyde. Results from quantum chemical modeling showed that formation of HMS and HMSi was several orders of magnitude faster than that of their corresponding conjugate acids, HMSA and HMHSi. In addition, water involvement can largely accelerate respectively the formation rate of HMS/HMSA and HMSi, but decelerate that of HMHSi, demonstrating the non-negligible role of water in the formation process. Furthermore, our kinetic model implemented with the calculated parameters indicates that HMSi/HMHSi but not HMS/HMSA can significantly alter the pH values of atmospheric aqueous aerosols and HMHSi is the most abundant species among HMS/HMSA and HMSi/HMHSi. Therefore, the newly-discovered pathway via HMSi/HMHSi formation should be of great concern and its kinetic parameters should be implemented in future models of atmospheric chemistry.

## Introduction

Sulfate is an important component of atmospheric aerosols, especially in polluted areas such as in China. While inorganic sulfate is relatively known to a certain extent, little is known regarding organosulfates in aerosols. Atmospheric sulfur-containing organic compounds such as hydroxymethanesulfonate (HMS, Fig. S1a) have been recently of great interest in atmospheric chemistry. The dissolution of sulfur dioxide forms bisulfite ions (R1–R3) which are then transformed to HMS by reacting with formaldehyde in atmospheric droplets through a well-established aqueous reaction (R4)^1^. Carbonyl compounds such as formaldehyde can compete with other aqueous oxidants (e.g., H_2_O_2_, OH) for oxidation of bisulfite ions, negatively affecting sulfate formation and potentially altering the properties of atmospheric particles (e.g., activity of atmospheric aqueous aerosols). Recent studies have shown that HMS formed during haze periods was likably interpreted as sulfate in previous field measurements and recommended that atmospheric aerosol models need to include reactions involving organic sulfate formation in order to accurately assess atmospheric organic and inorganic sulfate yields^2^. The HMS formation was thought to be the most important pathway for atmospheric organic sulfate through a five-member-ring transition state (Fig. S1b) by attacking the sulfur atom in bisulfite ions on the carbon atom in formaldehyde (R4). In aqueous medium, HMS equilibrates with its conjugate acid CH_2_(OH)SO_3_H (hydroxymethanesulfonic acid, HMSA) or its conjugate base CH_2_(O^−^)SO_3_^−^ (oxidomethanesulfonate, OMS). However, the former equilibrium is predominant in the pH range of atmospheric aqueous aerosols^1,3^.

R1 $${\rm{S}}{{\rm{O}}}_{{\rm{2}}}({\rm{g}})+{{\rm{H}}}_{{\rm{2}}}{\rm{O}}\to {\rm{S}}{{\rm{O}}}_{{\rm{2}}}\cdot {{\rm{H}}}_{{\rm{2}}}{\rm{O}}({\rm{a}}{\rm{q}})$$

R2 $${\rm{S}}{{\rm{O}}}_{2}\cdot {{\rm{H}}}_{2}{\rm{O}}\rightleftharpoons {{\rm{H}}}_{2}{\rm{S}}{{\rm{O}}}_{3}$$

R3 $${{\rm{H}}}_{2}{\rm{S}}{{\rm{O}}}_{3}+{{\rm{H}}}_{2}{\rm{O}}\rightleftharpoons {\rm{H}}{\rm{S}}{{\rm{O}}}_{3}^{-}+{{\rm{H}}}_{3}{{\rm{O}}}^{+}$$

R4 $${\rm{H}}{\rm{S}}{{{\rm{O}}}_{3}}^{-}+{\rm{H}}{\rm{C}}{\rm{H}}{\rm{O}}\rightleftharpoons {\rm{C}}{{\rm{H}}}_{2}({\rm{O}}{\rm{H}}){\rm{S}}{{{\rm{O}}}_{3}}^{-}({\rm{H}}{\rm{M}}{\rm{S}})$$

In this report, we propose an alternative pathway for the reaction between bisulfite ions and formaldehyde under aqueous environments, that is, the carbon atom in formaldehyde can be attacked by an oxygen atom instead of the sulfur atom in bisulfite ions (R5). This reaction proceeds via a proposed six-member-ring transition state (Fig. S1d), leading to formation of an HMS isomer, hydroxymethyl sulfite (HMSi, CH_2_(OH)OSO_2_^−^, Fig. S1c).

R5 $${\rm{H}}{\rm{S}}{{{\rm{O}}}_{3}}^{-}+{\rm{H}}{\rm{C}}{\rm{H}}{\rm{O}}\rightleftharpoons {\rm{C}}{{\rm{H}}}_{2}({\rm{O}}{\rm{H}}){\rm{O}}{\rm{S}}{{{\rm{O}}}_{2}}^{-}({\rm{H}}{\rm{M}}{\rm{S}}{\rm{i}})$$

Similarly, HMSi equilibrates with its conjugate acid CH_2_(OH)OSO_2_H (hydroxymethyl hydrogen sulfite, HMHSi) or its conjugate base CH_2_(O^−^)OSO_2_^−^ (oxidomethyl sulfite, OMSi), depending on the pH value in aqueous medium. Similar to HMS, HMHSi exists as the major conjugate compound in atmospheric aqueous aerosols.

**Figure 1:**
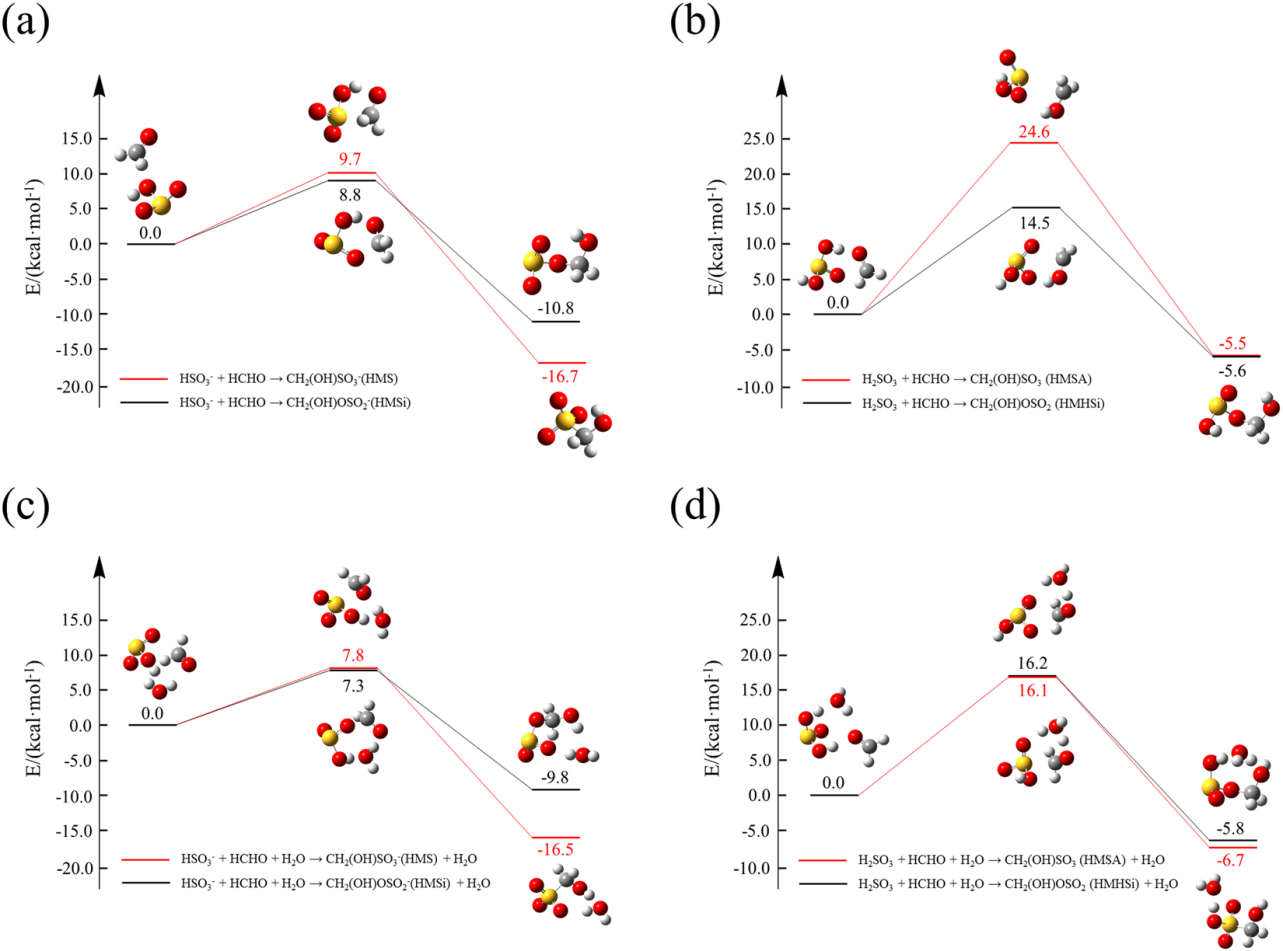
Potential energy profiles for the reactions (R4-R7): (**a**) and (**b**) show the generation pathways of HMS (R4) and HMSi (R5) without water involvement, while (**c**) and (**d**) show the water-involved generation pathways of HMS (R6) and HMSi (R7). All the energies are corrected with the zero-point energy (ZPE).

Here we performed quantum-chemical modeling to kinetically and thermodynamically evaluate the formation of HMS/HMSA and HMSi/HMHSi. As water can transform protons and lead to hydrogen-shift in aqueous environment, the reactions R4/R5 become R6/R7 respectively by including water into the reaction systems (Figs. 1a,b) to explore the effects of water as solvent on the kinetics of the reactions. Reaction rate constants were calculated to kinetically compare the respective pathways for the HMS and HMSi formation. The proton-dissociated equilibrium constants for the conjugate acids HMSA/HMHSi were calculated to evaluate their effects on pH values in atmospheric aqueous aerosols through our “in-house” kinetic model.

R6 $${\rm{H}}{\rm{S}}{{{\rm{O}}}_{3}}^{-}+{\rm{H}}{\rm{C}}{\rm{H}}{\rm{O}}+{{\rm{H}}}_{2}{\rm{O}}\rightleftharpoons {\rm{C}}{{\rm{H}}}_{2}({\rm{O}}{\rm{H}}){\rm{S}}{{{\rm{O}}}_{3}}^{-}({\rm{H}}{\rm{M}}{\rm{S}})+{{\rm{H}}}_{2}{\rm{O}}$$

R7 $${\rm{H}}{\rm{S}}{{{\rm{O}}}_{3}}^{-}+{\rm{H}}{\rm{C}}{\rm{H}}{\rm{O}}+{{\rm{H}}}_{2}{\rm{O}}\rightleftharpoons {\rm{C}}{{\rm{H}}}_{2}({\rm{O}}{\rm{H}}){\rm{O}}{\rm{S}}{{{\rm{O}}}_{2}}^{-}({\rm{H}}{\rm{M}}{\rm{S}}{\rm{i}})+{{\rm{H}}}_{2}{\rm{O}}$$

## Methods

### Quantum chemical modeling

Different isomers were generated to explore the global-minimum structures through molecular dynamic (MD) simulations at 298.15 K in a 10 nm × 10 nm × 10 nm box using the canonical ensemble (NVT). All the MD simulations were carried out using Large-scale Atomic/Molecular Massively Parallel Simulator (LAMMPS) package^4^ with the charge equilibration (QEq) scheme proposed by Rappe and Goddard^5^. The MD simulations employed the reactive force field (ReaxFF)^6^ whose parameters are implemented in LAMMPS and can be directly used without solvation model. The initial structures were then geometrically optimized at PM6 level, and subsequently refined by the B3LYP/6–31 G(d,p) method with the implicit polarizable continuum model, SMD^7^. The B3LYP/6–31 G(d,p) was also used to perform intrinsic reaction coordinate (IRC)^8^ calculations to verify the transition states. All the quantum-chemical modeling was performed using Gaussian 16 program^9^.

The reaction rate constant (k) was calculated by canonical transition state theory (CTST)

$${\rm{k}}={\rm{\kappa }}{{\rm{\nu }}}^{\ne }{\rm{\exp }}\left(\frac{-\Delta {{\rm{G}}}^{\ne }}{{{\rm{k}}}_{{\rm{B}}}{\rm{T}}}\right),$$

where

$$\Delta {{\rm{G}}}^{\ne }=\Delta {{\rm{E}}}^{{\rm{S}}{\rm{P}}}+\Delta {{\rm{g}}}^{{\rm{corr}}},$$

$${\rm{\kappa }}=1+\frac{1}{24}{\left(\frac{{\rm{h}}{{\rm{\nu }}}^{\ne }}{{{\rm{k}}}_{{\rm{B}}}{\rm{T}}}\right)}^{2}.$$

In the equations, κ is the transmission coefficient corresponding to the quantum tunneling^10^, ν^≠^ is the imaginary vibrational frequency of the transition state, ΔG^≠^ is the difference of Gibbs free energy between the transition state and the ground state. E^SP^ is the single-point energy, g^corr^ is the thermo correction of Gibbs free energy, k_B_ is the Boltzmann constant, h is the Planck’s constant and T is the temperature in Kelvin. To obtain an accurate result, the B3LYP functional with Grimme’s D3BJ dispersion calibration^11^ was used to optimize the transition-state structures and calculate imaginary vibrational frequency of transition states (ν^≠^) due to its good performance in vibrational analysis^12^. For comparison, two basis sets, 6–311++G(3df,3pd) and def2-TZVPPD were used for an accurate calculation. The M06–2X/def2-TZVP method was used to select the structure with the global minimum energy, and further optimization and vibrational analysis were performed with the M06-2X functional due to its good performance in thermodynamic calculations^13,14^, implemented with the Grimme’s D3 dispersion calibration^15^ and the basis set 6–311++G(3df,3pd), and hence the thermo correction to Gibbs free energy (g^corr^) was obtained. Zero-point energy (ZPE) correction for the energy barriers was also obtained by the same method. Single point energy (E^SP^) was calculated with the CCSD(T)/aug-cc-pVTZ method.

We firstly calculated the activation barriers and reaction rate constants for the formation of HMS/HMSA and HMSi/HMHSi without and with the involvement of water respectively. We then developed a chemical kinetic model to quantitatively assess how HMS/HMSA and HMSi/HMHSi alter the acidity of atmospheric aqueous aerosols. The implicit solvation model SMD^7^ was employed to include the effects of solvation in aqueous medium. All the relevant structures and calculated kinetic/thermodynamic parameters (Tables S1–S3) were included in the Supporting Information (SI).

### Kinetic modeling

Our “in-house” kinetic model includes the uptake of SO_2_, eleven aqueous reactions, and five chemical equilibria (SR1-SR16 in SI). The SO_2_ concentration, initial pH value, and particulate diameter were set as input parameters before the simulations, while output from the simulations included the pH values and the concentrations of the reaction products (i.e., HMS, HMSA, HMSi, HMHSi, etc). The uptake rate of SO_2_ onto the droplet was formulated as ${{\rm{R}}}_{{\rm{S}}{{\rm{O}}}_{2}}^{{\rm{u}}{\rm{p}}{\rm{t}}{\rm{a}}{\rm{k}}{\rm{e}}}=\frac{1}{4}\gamma [{\rm{S}}{{\rm{O}}}_{2}]{{\rm{v}}}_{{\rm{s}}{\rm{m}}{\rm{r}}}As$, where γ is the uptake coefficient of SO_2_ on a water droplet, v_smr_ is the square-mean-root velocity of SO_2_, [SO_2_] is the concentration of SO_2_ in the air, and As is the surface area of the droplet. The rates of the remaining reactions follow rate law and the steepest descent method is used to solve the nonlinear equations with a convergence limit of 1.0 × 10^−3^. Concentrations of the relevant species were then calculated after the simulations were successfully run. Additional detailed description of the kinetic model could be found in the SI.

## Results and Discussion

### Reactions of bisulfite ions or sulfurous acid with formaldehyde

The activation barriers for the formation of HMS/HMSi are 9.7 and 8.8 kcal mol^−1^ respectively (Fig. 1a), suggesting that the HMSi formation is kinetically more favorable than HMS. The calculated forward reaction rate constants are 1.5 × 10^4^ Lmol^−1^s^−1^ (R4) and 4.2 × 10^4^ Lmol^−1^s^−1^ (R5), while the corresponding dissociation rate constants (5.3 × 10^−2^ s^−1^, R4) and (1.6 × 10^−6^ s^−1^, R5) are relatively small. Hence the formation of HMSi is faster but much more unstable than that of HMS, suggesting that the concentration of HMSi may reach its peak value earlier than that of HMS. A previous study reported an experimental rate constant of 790 Lmol^−1^s^−1^ for the HMS formation^1^, about two orders of magnitude smaller than our calculated value. This bias indicates differences between measured and calculated rate constants. In fact, experiments can only provide comprehensive rate constants that include many reactions and the calculated rate constants are determined from specific reactions, probably only some of the above mentioned reactions in experiments, implying that other slower reaction pathways may exist and play non-negligible roles. We proposed that possible pathways (R8–R11) exist for reactions involving sulfurous acid (H_2_SO_3_) with formaldehyde via similar mechanisms to sulfite ions, leading respectively to the formation of HMSA/HMHSi since H_2_SO_3_ is a weak acid and exists in a large quantity under aqueous environments.

R8 $${{\rm{H}}}_{2}{\rm{S}}{{\rm{O}}}_{3}+{\rm{H}}{\rm{C}}{\rm{H}}{\rm{O}}\rightleftharpoons {\rm{C}}{{\rm{H}}}_{2}({\rm{O}}{\rm{H}}){\rm{S}}{{\rm{O}}}_{3}{\rm{H}}({\rm{H}}{\rm{M}}{\rm{S}}{\rm{A}})$$

R9 $${{\rm{H}}}_{2}{\rm{S}}{{\rm{O}}}_{3}+{\rm{H}}{\rm{C}}{\rm{H}}{\rm{O}}\rightleftharpoons {\rm{C}}{{\rm{H}}}_{2}({\rm{O}}{\rm{H}}){\rm{O}}{\rm{S}}{{\rm{O}}}_{2}{\rm{H}}({\rm{H}}{\rm{M}}{\rm{H}}{\rm{S}}{\rm{i}})$$

Results from similar quantum chemical modeling showed that the activation barriers are 24.6 and 14.5 kcal mol^−1^ for R8 and R9 respectively (Fig. 1b), significantly higher than those for R4 and R5. The calculated forward reaction rate constants are 1.4 × 10^−6^ and 9.0 Lmol^−1^s^−1^ for R8 and R9 respectively, far slower than those for R4 and R5. The formation of HMS/HMSA and HMSi/HMHSi may be decelerated by considering the equilibrium between HSO_3_^−^ and H_2_SO_3_. Note that the backward reaction rates are 7.0 × 10^−9^ and 2.7 × 10^−2^ s^−1^ respectively, indicating that HMSA can be more stable than HMHSi.

In general, water can catalyze aqueous reactions via forming cyclic ring transition states. Our calculations showed that the activation barriers are 7.8, 7.3, 16.2, and 16.1 kcal mol^−1^ respectively for R6, R7, R10, and R11 (Figs. 1c,d). The involvement of water can lower the activation barriers for R6, R7, and R10, while increase the barrier for R11 which is probably due to the steric instability of a large eight-member-ring transition state (Fig. 1d)^16^.

R10 $${{\rm{H}}}_{2}{\rm{S}}{{\rm{O}}}_{3}+{\rm{H}}{\rm{C}}{\rm{H}}{\rm{O}}+{{\rm{H}}}_{2}{\rm{O}}\rightleftharpoons {\rm{C}}{{\rm{H}}}_{2}({\rm{O}}{\rm{H}}){\rm{S}}{{\rm{O}}}_{3}{\rm{H}}({\rm{H}}{\rm{M}}{\rm{S}}{\rm{A}})+{{\rm{H}}}_{2}{\rm{O}}$$

R11 $${{\rm{H}}}_{2}{\rm{S}}{{\rm{O}}}_{3}+{\rm{H}}{\rm{C}}{\rm{H}}{\rm{O}}+{{\rm{H}}}_{2}{\rm{O}}\rightleftharpoons {\rm{C}}{{\rm{H}}}_{2}({\rm{O}}{\rm{H}}){\rm{O}}{\rm{S}}{{\rm{O}}}_{2}{\rm{H}}({\rm{H}}{\rm{M}}{\rm{H}}{\rm{S}}i)+{{\rm{H}}}_{2}{\rm{O}}$$

### Kinetic studies

The results from this study have important implications for the effects of reaction products on the acidity of atmospheric aqueous aerosols especially during haze events when concentrations of both sulfur dioxide and formaldehyde are high. Here we develop a chemical kinetic model using the results from our quantum chemical modeling to simulate the effects of HMS/HMSA and HMSi/HMHSi formation on the aerosols (refer to SI for the reaction scheme). In the simulations, we made the following assumptions based on the concentrations during a typical haze event: the droplet diameter = 2.5 μm, temperature = 298.15 K, the pressure = 1.0 atm, [SO_2_] = 50 ppb, the total amount of formaldehyde and methanediol in the droplet = 5.0 × 10^−5^ μM^17,18^, the uptake coefficient^19^ of SO_2_ adsorption = 5.0 × 10^−3^, initial pH = 5.0. The initial concentrations of all the products (i.e. HMS, HMSi, HMSA, and HMHSi) are set to be zero in our simulations. We performed the kinetic simulations based on four scenarios shown below.

**Figure 2:**
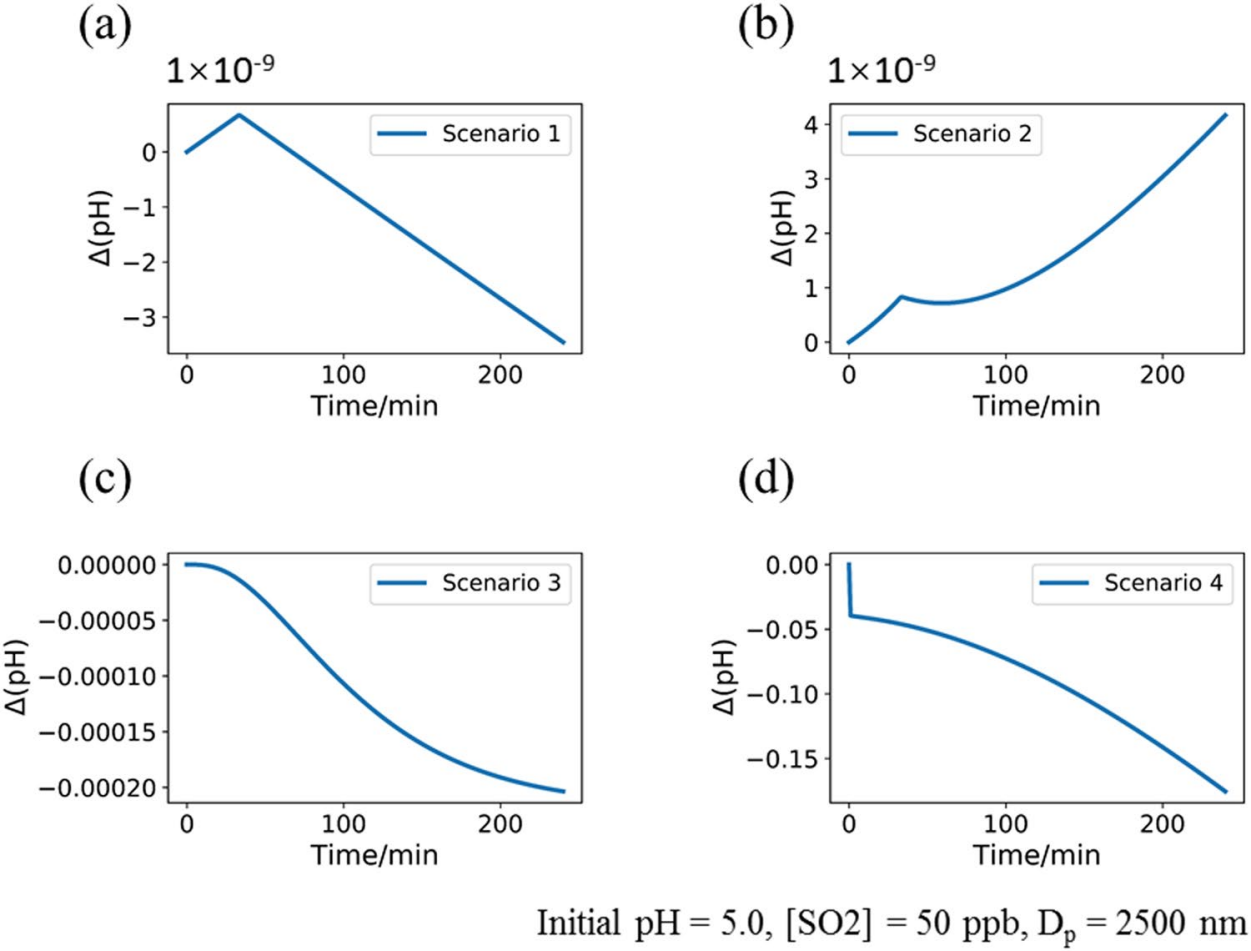
The changes of the pH value for the proposed four scenarios (**a**–**d**, see text for details). The initial pH is set to be 5.0, the concentration of SO_2_ is 50 ppb, and the particulate diameter is 2500 nm.

The first two scenarios considered only the reaction pathways for HMS formation (R4 and R6). The first one considered only the HMS formation with the reaction rate constant taken from Boyce and Hoffmann (Boyce and Hoffmann 1984); the second one considered both the HMS formation and dissociation with the reaction rate constants calculated in our study. A calculated equilibrium constant (K_HMS/HMSA_) of 8.52 × 10^−8^ for HMS/HMSA was used in the simulations. The results showed different trends of pH changes in the later period of the two scenarios (Figs. 2a,b), implying that the stability of the HMS or HMSi can either increase or decrease the acidity of the aerosols, although the impacts are not significant (only up to 4 × 10^−9^ for the changes in pH values during a reaction time of about four hours). Hence neither case could exert significant impacts on the pH values of the droplets.

**Figure 3:**
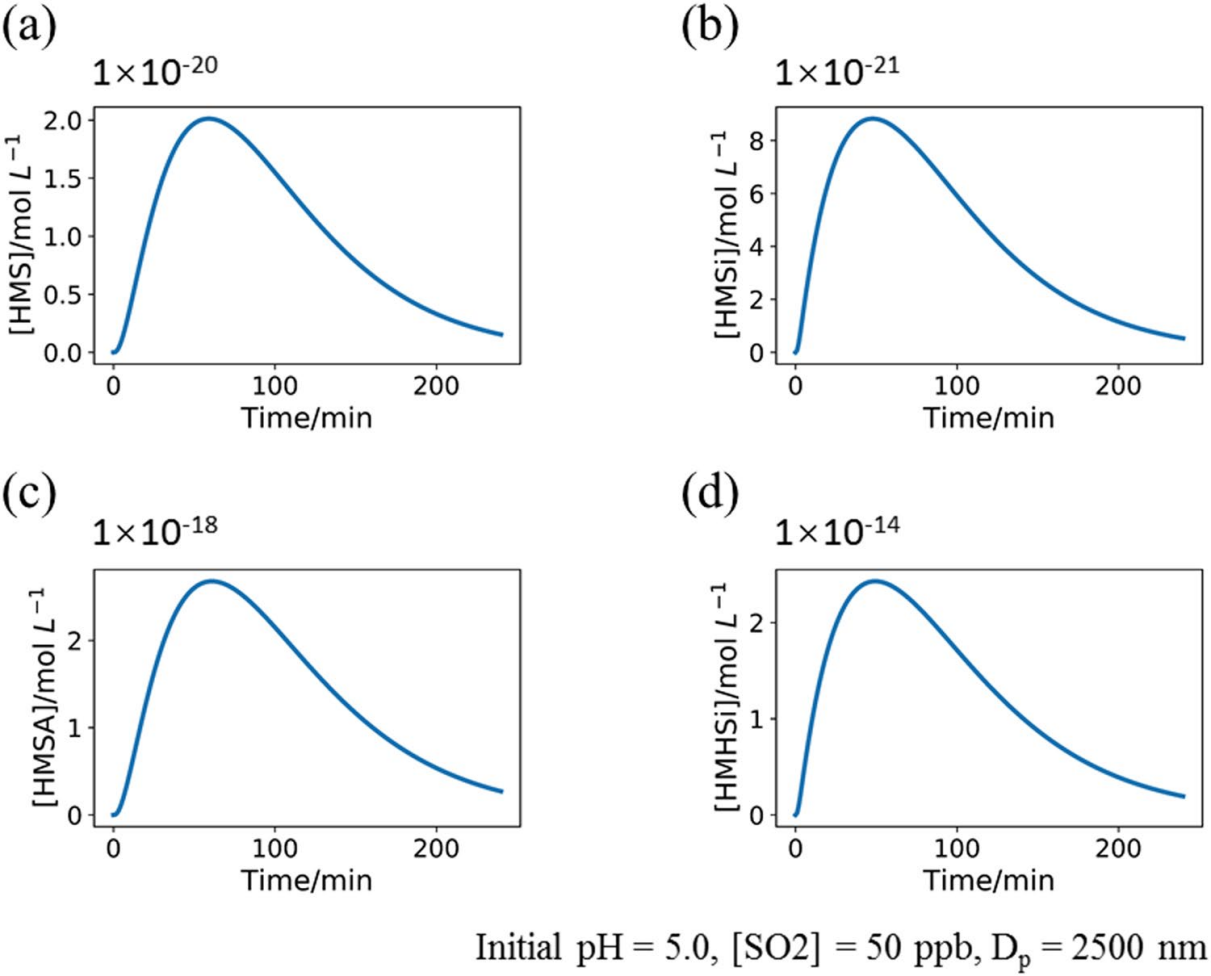
The concentration of (**a**) HMS, (**b**) HMSi, (**c**) HMSA, (**d**) HMHSi produced during the reaction period. The initial pH is set to be 5.0, the concentration of SO_2_ is 50 ppb, and the particulate diameter is 2500 nm.

The third scenario considered the formation and dissociation of HMSA (R8 and R10) in the simulation which can decrease the pH values larger than those in the first scenario (up to about 4 × 10^−4^ for the changes in pH values during a reaction time of about four hours as shown Fig. 2c), indicating the significance of direct reactions between H_2_SO_3_ and formaldehyde. The fourth scenario considered all the reactions involving HMSi and HMHSi (R5, R7, R9, and R11) and an equilibrium constant (K_HMSi/HMHSi_) of 4.08 × 10^−12^ between HMSi and HMHSi was employed in the simulations. The results showed that the pH value momentarily decreased for nearly 0.05 in the very beginning of the simulations and continued to decrease at a slower rate afterward up to a pH change of about 0.2 (Fig. 2d). The concentrations of HMS, HMSA, HMSi and HMHSi varied in a similar way: initially increased to a maximum at a reaction time of about one hour and subsequently decreased at a slower rate than the initial rising rate. In addition, the peak concentration of HMHSi is far higher than all others (Fig. 3), corresponding to a range of 0.3–3.8 ng m^−3^ in the aerosol masses (Table S6), within the range of the reported concentration (0.3–38.5 ng m^−3^) of HMS in clouds^20^. This may imply that a large fraction of HMS identified in previous studies is probably attributed to HMHSi instead. Note that the concentration of HMSi was found to be low due to the fact that K_HMSi/HMHSi_ is several orders of magnitude smaller than K_HMS/HMSA_, leading to the transformation of a large fraction of HMSi into HMHSi via the ionization equilibrium.

### Sensitivity studies

**Figure 4:**
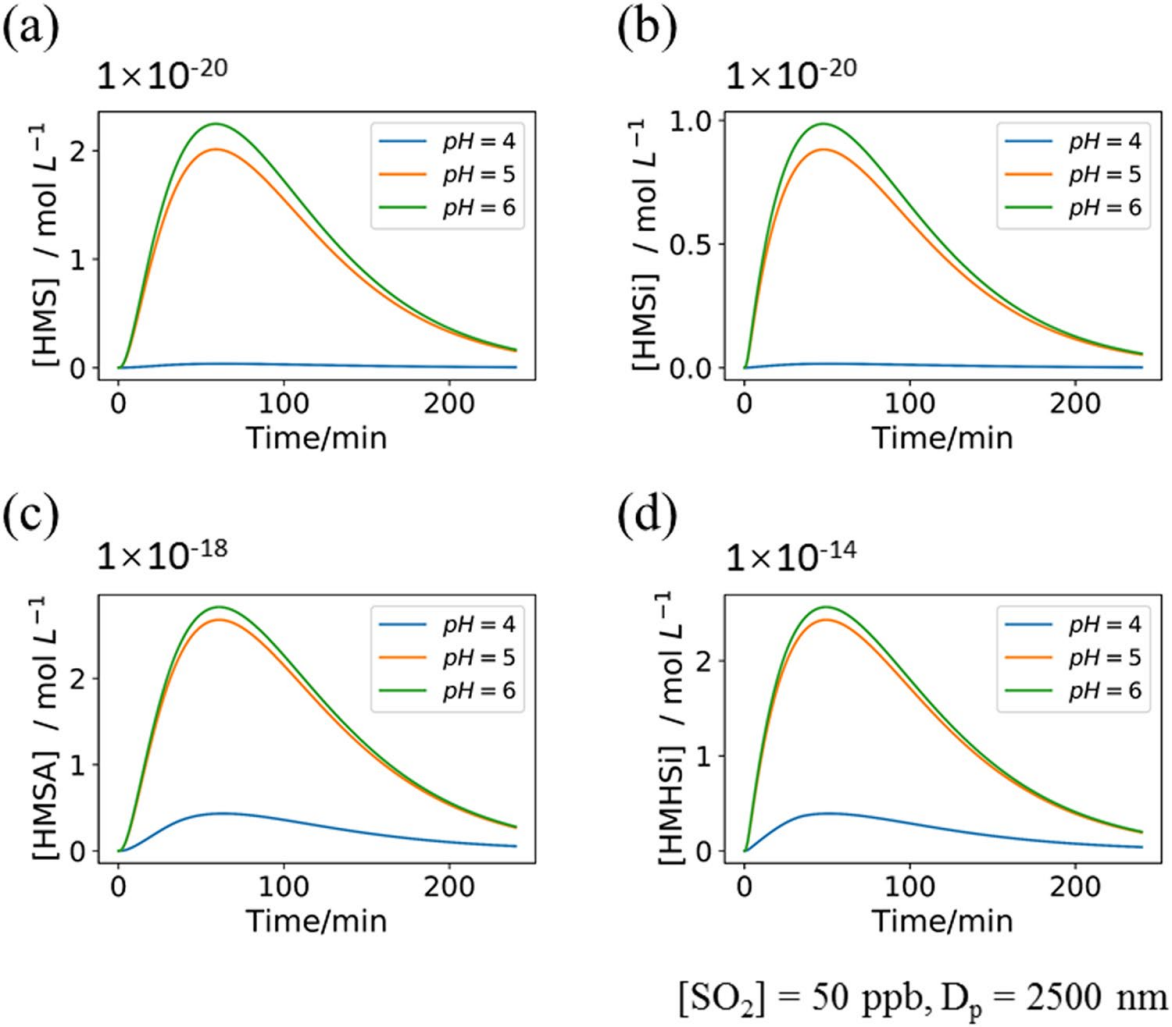
The concentration of (**a**) HMS, (**b**) HMSi, (**c**) HMSA, (**d**) HMHSi with different initial pH values. The concentration of SO_2_ is 50 ppb and the particulate diameter is 2500 nm.

In addition, sensitivity studies were performed to validate the applicability of the proposed kinetic model. Three cases were tested: initial pH value (4.0, 5.0, and 6.0; case one), particulate diameter (2.0 μm, 2.5 μm, and 3.0 μm; case two), and the SO_2_ concentration (40 ppb, 50 ppb, and 60 ppb; case three). Note that only the gaseous SO_2_ but not HCHO or CH_2_(OH)_2_ concentration was selected for sensitivity tests due to: (1) the SO_2_ concentration covers a broad range from ppt level in clean air to several tens ppb level in polluted atmosphere and thus affects the SO_2_ uptake in aqueous aerosols; (2) equilibria involving SO_2_ and H_2_SO_3_ (R2 and R3) can directly influence the pH values. The results from sensitivity studies showed that most of our simulations are robust (Figs. S2–S19 in SI). However, the concentrations of HMS, HMSi, HMSA and HMHSi are extremely sensitive to the initial pH values between 4.0 and 5.0 (Fig. 4, and Figs. S20–28 in SI). In addition, the change of the HSO_3_^−^ concentration has the same trend as that of the pH value (Figs. S29–30 in SI), but the H_2_SO_3_ concentration is shown to be independent of the initial pH value (Figs. S29–30) which can be attributed to its equilibrium with SO_2_•H_2_O. Our kinetic simulations demonstrate the strong dependence of formation of atmospheric sulfur-containing organic compounds (i.e., HMS, HMSi, HMSA, and HMHSi) on the initial pH values especially in acidic aqueous aerosols.

## Conclusions

The properties in particular the pH values of atmospheric aqueous aerosols were significantly affected in the presence of organic sulfur-containing compounds. How organic sulfate modulates the pH values of the aerosols is currently poorly understood due in part to the difficulties in measuring the pH values at the particle size levels. In recent years, previous misinterpretation of organic sulfate as inorganic sulfate has been found and corrected. The then attribution of organic sulfate primarily to HMS was based upon the measurements using Aerosol Mass Spectrometer which is capable of differentiating organic sulfate from inorganic sulfate but is yet difficult to discriminate isomeric identities, i.e., between HMS and HMSi. Here we employed quantum chemical calculation and kinetic modeling to investigate the reactions between sulfur dioxide and formaldehyde in atmospheric aqueous aerosols. The results showed clearly that much faster formation rate for HMSi than for HMS was found either without or with the presence of water. Our “in-house” kinetic model considering different concentration scenarios clearly showed that the concentration of HMSi conjugate acid (HMHSi) exists in the most abundant amount among HMS and HMSi and their corresponding conjugate acids, indicating that formation of HMSi predominates over its counterpart HMS. Combining results from both quantum chemical calculation and kinetic modeling, we concluded that the reactions between sulfur dioxide and formaldehyde in aqueous medium proceed predominantly via the HMSi/HMHSi pathway instead of previously reported HMS/HMSA one and highlighted the importance of HMHSi in modulating the acidity of atmospheric aqueous aerosols. It is hence that reaction mechanisms involving formation of HMSi/HMHSi should be included in atmospheric aerosol models in order to accurately assess the organic and inorganic sulfate aerosols in the atmosphere. Our study also demonstrates the potent ability for theoretical calculations to illustrate complicated mechanisms at the microscopic molecular level when the reaction systems are involved in isomers such as HMS and HMSi which otherwise current advanced instruments can hardly discriminate. In addition, our work might provide valuable information for further analysis of organosulfates in atmospheric aerosols in future studies.

## Supplementary information

Supplementary information.

## Data Availability

All the data presented in this work can be found in the supplementary information. The kinetic model developed in this work is available upon request from K.P.C. (kchen255@ucr.edu).
