# Supplementary material for: Theoretical investigation of a potentially important formation pathway of organosulfate in atmospheric aqueous aerosols
Source: Sci Rep. 2020 Apr 14;10:6299. doi: 10.1038/s41598-020-61968-2 (PMC7156694; doi:10.1038/s41598-020-61968-2)
Supplement: Supplementary file 1 — Supplementary information. [file 41598_2020_61968_MOESM1_ESM.pdf]

Supporting Information

Theoretical investigation of a potentially important formation pathway of organosulfate in atmospheric aqueous aerosols

Kunpeng Chen[1,4] and Jun Zhao[1,2,3*]

[1]School of Atmospheric Sciences, Sun Yat-sen University, Guangzhou, Guangdong, 510275, China

[2]Guangdong Province Key Laboratory for Climate Change and Natural Disaster Studies, and Institute of Earth Climate and Environment System, Sun Yat-sen University, Guangzhou, Guangdong, 510275, China

[3]Southern Laboratory of Ocean Science and Engineering (Guangdong, Zhuhai), Zhuhai, Guangdong, 519082, China

[4]Current affiliation: Department of Environmental Sciences, College of Natural and Agricultural Sciences, University of California Riverside, Riverside, CA, 92521, USA

[*]Corresponding author: Jun Zhao (zhaojun23@mail.sysu.edu.cn)]

Contents of this file

Text S1 to S2
Figures S1 to S30
Tables S1 to S6

1. The geometrical structures of hydroxymethanesulfonate (HMS), hydroxymethyl sulfite (HMSi), and their corresponding transition states of formation are shown in Figure S1.
2. Calculated imaginary vibrational frequencies for the relevant transition states at different levels of theory are given in Table S1.
3. Calculated thermo correction to Gibbs free energy and single point energy for the reactions is given in Table S2.
4. Calculated reaction rate constants at different levels of theory are given in Table S3.
5. Description of Calculations of $K_{HMS/HMSA}$ and $K_{HMSi/HMHSi}$ is given in Text S1.
6. Values of $K_{HMS/HMSA}$ and $K_{HMSi/HMHSi}$ are given in Table S4.
7. The XYZ coordinates of the structures of relevant species are given in Table S5.
8. Maximum concentration of HMHSi under different simulating conditions is given in Table S6.
9. Results of sensitivity studies are shown in Figures S2-S30.
10. Detailed description of the kinetic model is given in Text S2.

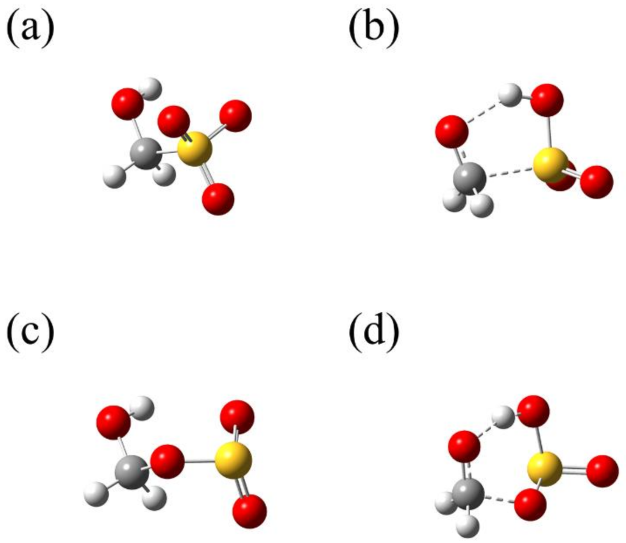

Figure S1. Geometrical structures of (a) HMS, (b) the five-member-ring transition state corresponding to HMS, (c) HMSi, (d) the six-member-ring transition state corresponding to HMSi. In the ball-bond view, the oxygen atoms are in red, the hydrogen atoms are in white, the carbon atoms are in gray, and the sulfur atoms are in yellow.

Table S1. Calculated imaginary vibrational frequencies for the relevant transition states at different levels of theory

Reaction	B3LYP-D3BJ/ 6-311++G(3df,3dp) cm^{-1}	B3LYP-D3BJ/ def2-TZVPPD cm^{-1}
H_2SO_3 + HCHO $\rightleftharpoons$ $CH_2(OH)SO_3H$ (HMSA)	-282.36	-287.99
H_2SO_3 + HCHO + H_2O $\rightleftharpoons$ $CH_2(OH)SO_3H$ (HMSA) + H_2O	-865.28	-830.54
H_2SO_3 + HCHO $\rightleftharpoons$ $CH_2(OH)OSO_2H$ (HMHSi)	-317.18	-321.57
H_2SO_3 + HCHO + H_2O $\rightleftharpoons$ $CH_2(OH)OSO_2H$ (HMHSi) + H_2O	-176.71	-189.35
HSO_3^- + HCHO $\rightleftharpoons$ $CH_2(OH)SO_3^-$ (HMS)	-216.46	-213.01
HSO_3^- + HCHO + H_2O $\rightleftharpoons$ $CH_2(OH)SO_3^-$ (HMS) + H_2O	-199.02	-196.00
HSO_3^- + HCHO $\rightleftharpoons$ $CH_2(OH)OSO_2^-$ (HMSi)	-294.08	-291.61
HSO_3^- + HCHO + H_2O $\rightleftharpoons$ $CH_2(OH)OSO_2^-$ (HMSi) + H_2O	-422.38	-428.47

Table S2. Calculated thermo correction to Gibbs free energy and single point energy for the reactions

Reaction	Reactants		TS		Products	
	g^{corr} /a.u.	E^{SP} /a.u.	g^{corr} /a.u.	E^{SP} /a.u.	g^{corr} /a.u.	E^{SP} /a.u.
HSO_3^- + HCHO $\rightleftharpoons$ $CH_2(OH)SO_3^-$ (HMS)	0.015	-738.254	0.021	-738.241	0.027	-738.288
HSO_3^- + HCHO $\rightleftharpoons$ $CH_2(OH)OSO_2^-$ (HMSi)	0.015	-738.254	0.021	-738.242	0.026	-738.278
H_2SO_3 + HCHO $\rightleftharpoons$ $CH_2(OH)SO_3H$ (HMSA)	0.028	-738.703	0.032	-738.666	0.038	-738.717
H_2SO_3 + HCHO $\rightleftharpoons$ $CH_2(OH)OSO_2H$ (HMHSi)	0.028	-738.703	0.032	-738.681	0.037	-738.717
HSO_3^- + HCHO + H_2O $\rightleftharpoons$ $CH_2(OH)SO_3^-$ (HMS) + H_2O	0.040	-814.620	0.042	-814.608	0.048	-814.651
HSO_3^- + HCHO + H_2O $\rightleftharpoons$ $CH_2(OH)OSO_2^-$ (HMSi) + H_2O	0.040	-814.620	0.043	-814.609	0.046	-814.640
H_2SO_3 + HCHO + H_2O $\rightleftharpoons$ $CH_2(OH)SO_3H$ (HMSA) + H_2O	0.048	-815.069	0.051	-815.042	0.057	-815.084
H_2SO_3 + HCHO + H_2O $\rightleftharpoons$ $CH_2(OH)OSO_2H$ (HMHSi) + H_2O	0.048	-815.069	0.050	-815.043	0.058	-815.083

Table S3. Calculated reaction rate constants at different levels of theory

Reaction	k (B3LYP-D3BJ/6-311++G(3df,3pd))*		k (B3LYP-D3BJ/def2-TZVPPD)*	
	$k_{forward}$ / $Lmol^{-1}s^{-1}$	$k_{backward}$ / s^{-1}	$k_{forward}$ / $Lmol^{-1}s^{-1}$	$k_{backward}$ / s^{-1}
HSO_3^- + HCHO ⇌ $CH_2(OH)SO_3^-$(HMS)	1.54×10^{4}	1.56×10^{-6}	1.51×10^{4}	1.53×10^{-6}
HSO_3^- + HCHO ⇌ $CH_2(OH)OSO_2^-$(HMSi)	4.23×10^{4}	5.29×10^{-2}	4.19×10^{4}	5.24×10^{-2}
H_2SO_3 + HCHO ⇌ $CH_2(OH)SO_3H$(HMSA)	1.37×10^{-6}	7.04×10^{-9}	1.40×10^{-6}	7.20×10^{-9}
H_2SO_3 + HCHO ⇌ $CH_2(OH)OSO_2H$(HMHSi)	8.97×10^{0}	2.73×10^{-2}	9.12×10^{0}	2.78×10^{-2}
HSO_3^- + HCHO + H_2O ⇌ $CH_2(OH)SO_3^-$(HMS) + H_2O	1.01×10^{6}	2.30×10^{-5}	9.92×10^{5}	2.26×10^{-5}
HSO_3^- + HCHO + H_2O ⇌ $CH_2(OH)OSO_2^-$(HMSi) + H_2O	6.25×10^{6}	1.46×10^{0}	6.37×10^{6}	1.49×10^{0}
H_2SO_3 + HCHO + H_2O ⇌ $CH_2(OH)SO_3H$(HMSA) + H_2O	1.53×10^{0}	9.93×10^{-4}	1.42×10^{0}	9.21×10^{-4}
H_2SO_3 + HCHO + H_2O ⇌ $CH_2(OH)OSO_2H$ (HMHSi) + H_2O	1.05×10^{0}	4.69×10^{-3}	1.13×10^{0}	5.04×10^{-3}

*Single point energies were calculated at CCSD(T)/aug-cc-pVTZ level; frequency calculations were performed at the respective level of theory as indicated in the table.

Text S1

Calculations of $K_{HMS/HMSA}$ and $K_{HMSi/HMHSi}$

We term HA as the weak acid (i.e. HMSA and HMHSi) and A^- as the ionized product (i.e. HMS and HMSi) for the equilibrium

$$HA + H_2O \rightleftharpoons A^- + H_3O^+$$

The equilibrium constant ($K_{A^-/HA}$) can be calculated as

$$K_{A^-/HA} = \frac{(c_{A^-}/c_0)(c_{H_3O^+}/c_0)}{(c_{HA}/c_0)} = \exp\left(-\frac{\Delta G^{eq}}{RT}\right),$$

where c_0 is the unit concentration (1.0 mol L^{-1}), and c_{A^-}, $c_{H_3O^+}$, c_{HA} is the concentration of A^-, H_3O^+ and HA, respectively. The concentration is normalized to obtain a unitless equilibrium constant.

$$\Delta G^{eq} = (G_{f,A^-} + G_{f,H_3O^+}) - (G_{f,HA} + G_{f,H_2O}),$$

$$G_f = g^{corr} + E^{SP},$$

where ΔG^{eq} is the Gibbs free energy difference between the right-hand side and left-hand side of the equilibrium, and G_f is the formation Gibbs free energy which can be decomposed into the thermo-correction of Gibbs free energy (g^{corr}) and the single-point energy (E^{SP}). Relevant data is summarized in Table S4. Our calculations show that $K_{HMS/HMSA}$ and $K_{HMSi/HMHSi}$ are equal to 8.52×10^{-8} and 4.08×10^{-12}, respectively.

Table S4. The calculated thermo correction and the single point energy for the relevant compounds

Compound	HMS	HMSA	HMSi	HMHSi	H_2O	H_3O^+
g^{corr} / a.u.	0.027	0.038	0.026	0.037	0.004	0.016
E^{SP} / a.u.	-738.288	-738.717	-738.278	-738.717	-76.354	-76.770
G_f / a.u.	-738.261	-738.680	-738.252	-738.680	-76.350	-76.754

Table S5. The XYZ coordinates of the structures of the relevant species

(1) HSO_3^- + HCHO → $CH_2(OH)SO_3^-$ (HMS)

HSO_3^- + HCHO

9

C	2.28210	0.59920	0.27060
O	2.95040	0.08610	-0.59370
H	1.72380	-0.00110	0.99860
H	2.22660	1.69020	0.37850
S	-1.15210	-0.12650	0.01010
O	-1.00790	0.25580	1.43600
O	-0.31790	-1.30560	-0.31090
O	-0.21130	1.05070	-0.75370
H	-0.51260	1.91870	-0.45260

TS1

9

C	1.58020	-0.79250	-0.19190
O	2.25140	0.23860	0.07730
S	-0.54460	-0.13560	-0.00660
O	-1.50300	-0.30600	-1.10140
O	-1.13840	-0.35370	1.31540
O	-0.17560	1.44100	-0.05440
H	1.48110	-1.59450	0.54950
H	1.47840	-1.12780	-1.23140
H	0.79800	1.48710	0.04460

$CH_2(OH)SO_3^-$ (HMS)

9

C	1.09250	0.78820	-0.05240
O	2.15510	-0.11620	0.11830
S	-0.49640	-0.04010	0.00350
O	-0.62750	-0.61780	1.33890
O	-1.47240	1.01330	-0.26550
O	-0.43710	-1.05620	-1.04700

H	1.13840	1.31370	-1.00880
H	1.11450	1.50610	0.76620
H	2.18950	-0.69140	-0.65730

(2) HSO_3^- + HCHO → $CH_2(OH)OSO_2^-$ (HMSi)

HSO_3^- + HCHO

The structure is available in Table S5 (1).

TS2

9

O	0.39040	1.25100	-0.66830
C	-1.79470	-0.38410	-0.09370
H	-1.99910	-0.21660	-1.15560
H	-2.24600	-1.30080	0.29450
O	-1.71050	0.62940	0.67470
O	-0.21840	-1.09540	-0.37920
H	-0.45760	1.32660	-0.13780
S	1.03180	-0.18880	-0.28650
O	1.41190	-0.08450	1.13410

$CH_2(OH)OSO_2^-$ (HMSi)

9

O	-0.42600	1.36430	-0.30130
C	1.44590	-0.55320	0.44500
H	0.97290	-0.24630	1.38110
H	2.08440	-1.41750	0.61460
O	2.26010	0.47510	-0.06400
O	0.45650	-0.93620	-0.48830
H	1.68070	1.24570	-0.15990
S	-0.96570	-0.02120	-0.42450
O	-1.55500	-0.42670	0.87440

(3) H_2SO_3 + HCHO → $CH_2(OH)SO_3H$ (HMSA)

H_2SO_3 + HCHO

10

C	-5.13760	6.57040	3.84770
H	-4.07990	6.39240	4.07190
H	-5.63260	7.41920	4.32920
O	-5.74960	5.85410	3.08810
S	-2.96790	3.81330	2.47710
O	-3.15970	3.69190	4.08140
O	-2.44830	5.16820	2.36120
O	-4.47650	3.83660	2.02460
H	-3.39590	2.78440	4.33290
H	-4.97620	4.61880	2.42220

TS3

10

C	-2.06660	-0.51050	-0.63800
H	-2.33640	-0.43270	-1.68500
H	-1.75720	-1.44730	-0.18960
O	-2.23780	0.50630	0.06460
S	0.66270	-0.21980	-0.08450
O	0.09530	0.03230	1.26430
O	1.84600	-1.09210	-0.03410
O	1.28850	1.28120	-0.38110
H	-1.84300	0.39530	0.96450
H	1.77730	1.26180	-1.21670

$CH_2(OH)SO_3H$ (HMSA)

10

C	1.13210	0.62820	-0.45020
H	1.10080	0.76840	-1.52820
H	1.36340	1.56910	0.05030
O	2.00130	-0.41300	-0.13080
S	-0.53550	0.20750	0.05030
O	-0.53510	-0.12490	1.44440
O	-1.43940	1.20180	-0.43690
O	-0.84380	-1.08880	-0.78000
H	2.24790	-0.34750	0.79930
H	-0.40880	-1.87190	-0.40130

(4) H_2SO_3 + HCHO → $CH_2(OH)OSO_2H$ (HMHSi)

H_2SO_3 + HCHO

The structure is available in Table S5 (3).

TS4

10

O	-0.24180	-0.92680	-0.97170
C	2.01280	0.37490	0.06160
H	1.93320	0.59520	-0.99440
H	2.54230	1.04860	0.72570
O	1.71020	-0.75870	0.50030
O	0.02760	1.28930	0.02220
H	1.00270	-1.14760	-0.16530
S	-0.97400	0.25650	-0.38620
O	-1.37430	-0.28870	1.11370
H	-2.09440	-0.93330	1.04370

$CH_2(OH)OSO_2H$ (HMHSi)

10

O	-0.52580	1.30900	0.04940
C	1.45560	-0.65860	0.31780
H	0.95680	-0.56570	1.28110
H	2.03490	-1.57280	0.26930
O	2.29920	0.41330	0.06210
O	0.46350	-0.79360	-0.72540
H	1.84350	1.22820	0.30470
S	-0.89350	0.04950	-0.58750
O	-1.66270	-0.79530	0.53020
H	-1.33370	-0.61010	1.42840

(5) HSO_3^- + HCHO + H_2O → $CH_2(OH)SO_3^-$ (HMS) + H_2O

HSO_3^- + HCHO + H_2O

12

C	-1.99700	-1.05560	-0.36780
O	-2.47960	-0.35490	0.49210
H	-2.01960	-2.14830	-0.29160
H	-1.55300	-0.62490	-1.27290
O	-1.05530	2.02470	-0.17270

H	-1.72670	1.36920	0.07120
H	-0.45150	1.54360	-0.76560
H	0.29870	1.15470	1.01260
O	0.57390	-1.49080	0.15000
O	1.10950	0.65860	1.23550
S	1.49460	-0.33910	-0.03320
O	1.04150	0.47040	-1.20700

TS5

12

C	-0.06540	1.36820	-0.00300
O	-1.18500	1.23120	0.61160
S	0.93920	-0.53720	-0.07450
O	2.28570	-0.50570	-0.72850
O	0.91230	-1.22380	1.25630
O	0.00740	-1.41740	-1.08410
H	0.75910	1.89960	0.50000
H	-0.03810	1.47470	-1.10110
H	-0.99270	-1.05440	-1.04760
H	-2.02870	0.30240	-0.29820
O	-2.29890	-0.45840	-0.92810
H	-2.85670	-1.04320	-0.39380

$CH_2(OH)SO_3^-$ (HMS) + H_2O

12

C	0.13010	1.00870	0.02370
O	-1.02900	0.88250	0.80380
S	0.97020	-0.56190	-0.20760
O	2.13970	-0.24230	-1.02080
O	1.30630	-1.06390	1.12080
O	0.01690	-1.43510	-0.90690
H	0.83690	1.66220	0.53450
H	-0.07490	1.40030	-0.97590
H	-1.73020	0.51370	0.23350
H	-1.73130	-0.89090	-1.23150
O	-2.55760	-0.37590	-1.17880
H	-3.20600	-0.97630	-0.79280

(6) HSO_3^- + HCHO + H_2O → $CH_2(OH)OSO_2^-$ (HMSi) + H_2O

HSO_3^- + HCHO + H_2O

The structure is available in Table S5 (5).

TS6

12

C	1.15990	-1.37730	0.35160
O	2.05720	-0.77690	-0.30580
O	1.63170	1.66580	0.37640
H	0.18410	1.42920	-0.35900
O	-0.44430	-1.23050	-0.47600
O	-0.66060	1.20130	-0.85110
H	0.83240	-0.95980	1.30720
S	-1.41050	-0.06860	-0.19800
O	-1.39420	0.20560	1.25530
H	1.94290	0.74970	0.10260
H	2.14910	2.28700	-0.14540
H	1.11040	-2.46660	0.29970

$CH_2(OH)OSO_2^-$ (HMSi) + H_2O

12

C	0.90300	-1.36930	0.25530
O	1.98710	-0.82140	-0.44590
O	1.73470	1.79730	0.53870
H	0.84720	1.65510	0.14390
O	-0.28950	-1.35630	-0.50940
O	-0.56730	1.10830	-0.69510
H	0.74690	-0.84310	1.19770
S	-1.36830	-0.08560	-0.29240
O	-1.56590	-0.06110	1.17520
H	2.05200	0.11990	-0.21180
H	2.15750	2.45820	-0.01820
H	1.12480	-2.41610	0.44490

(7) H_2SO_3 + HCHO + H_2O → $CH_2(OH)SO_3H$ (HMSA) + H_2O

H_2SO_3 + HCHO + H_2O

13

C	-0.67930	-2.69420	-0.45380

O	-0.79020	-1.49010	-0.45870
H	0.25150	-3.17550	-0.77200
O	-2.21150	0.80830	0.34170
H	-1.98400	-0.11970	0.17500
H	-2.40150	0.86330	1.28490
H	0.52130	-0.46870	-0.85720
O	1.23310	0.20290	-1.02760
O	1.01430	0.60340	1.37000
O	-0.06160	2.12270	-0.20540
H	-0.90720	1.60400	0.03260
S	1.25920	1.31790	0.11440
H	-1.50880	-3.33080	-0.13010

TS7

13

C	-0.16600	1.46780	0.02310
O	-1.21160	1.06730	0.57460
S	1.11280	-0.61100	-0.22730
O	2.46620	-0.86490	-0.72390
O	1.12200	-1.05620	1.33240
O	0.13140	-1.53610	-0.85830
H	0.59100	1.97430	0.61890
H	-0.12270	1.57590	-1.06140
H	-1.41960	-1.02240	-1.02170
H	-1.88990	0.34920	-0.21710
O	-2.23450	-0.44010	-0.97710
H	-2.96100	-0.96430	-0.61170
H	1.31180	-2.00660	1.41280

$CH_2(OH)SO_3H$ (HMSA) + H_2O

13

C	-1.64650	-0.37930	0.35160
O	-1.09380	-1.66070	0.33820
S	-0.46260	0.81760	-0.25620
O	-1.04610	2.12240	-0.12780
O	0.71060	0.72390	0.73400
O	-0.02070	0.39230	-1.55900
H	-1.87930	-0.09220	1.37430
H	-2.52830	-0.27510	-0.28090
H	-1.13090	-2.01090	-0.55960

H	1.38840	-0.03480	0.48550
H	3.16510	-0.95250	0.56730
O	2.30720	-1.10150	0.15170
H	2.47840	-1.11040	-0.79800

(8) H_2SO_3 + HCHO + H_2O → $CH_2(OH)OSO_2H$ (HMHSi) + H_2O

HSO_3^- + HCHO + H_2O

The structure is available in Table S5 (7).

TS8

13

C	1.28490	1.20590	0.78930
H	1.47550	0.56320	1.64160
O	1.93590	1.07940	-0.25450
O	2.56690	-1.24310	-0.25800
H	2.40900	0.08560	-0.26970
H	1.66710	-1.52570	-0.01240
O	-0.13990	-0.71790	0.50290
H	3.15500	-1.54880	0.44400
O	-1.13380	0.81020	-1.10920
H	0.59370	2.03940	0.87140
O	-2.36170	0.03980	0.87980
H	-3.21380	0.28230	0.49130
S	-1.33640	-0.43990	-0.34650

$CH_2(OH)OSO_2H$ (HMHSi) + H_2O

13

C	-1.19180	4.07360	2.30940
H	-2.13760	3.98730	2.83640
O	-1.37800	4.16710	0.93050
H	-2.06510	4.81790	0.74460
O	-0.59110	5.25030	2.85250
O	1.61330	4.17930	2.57800
H	-0.55610	3.21450	2.49640
O	1.11460	6.21320	1.33070
H	1.05860	5.55960	0.53980
S	1.01230	5.50260	2.72720
O	0.88570	4.57520	-0.61140

H	0.06570	4.16910	-0.28180
H	1.56210	3.89130	-0.54580

Table S6. Maximum concentration of HMHSi under different simulating conditions.

D_p / nm	[SO2] / ppb	Initial pH	$[HMHSi]_{max}$ / (ng m^{-3})
2000	40	4.0	0.4
2000	40	5.0	2.7
2000	40	6.0	2.9
2000	50	4.0	0.5
2000	50	5.0	3.3
2000	50	6.0	2.9
2000	60	4.0	0.6
2000	60	5.0	3.8
2000	60	6.0	3.0
2500	40	4.0	0.4
2500	40	5.0	2.3
2500	40	6.0	2.8
2500	50	4.0	0.4
2500	50	5.0	2.7
2500	50	6.0	2.9
2500	60	4.0	0.5
2500	60	5.0	3.2
2500	60	6.0	2.9
3000	40	4.0	0.3
3000	40	5.0	1.9
3000	40	6.0	2.8
3000	50	4.0	0.4
3000	50	5.0	2.3
3000	50	6.0	2.8
3000	60	4.0	0.4
3000	60	5.0	2.7
3000	60	6.0	2.9

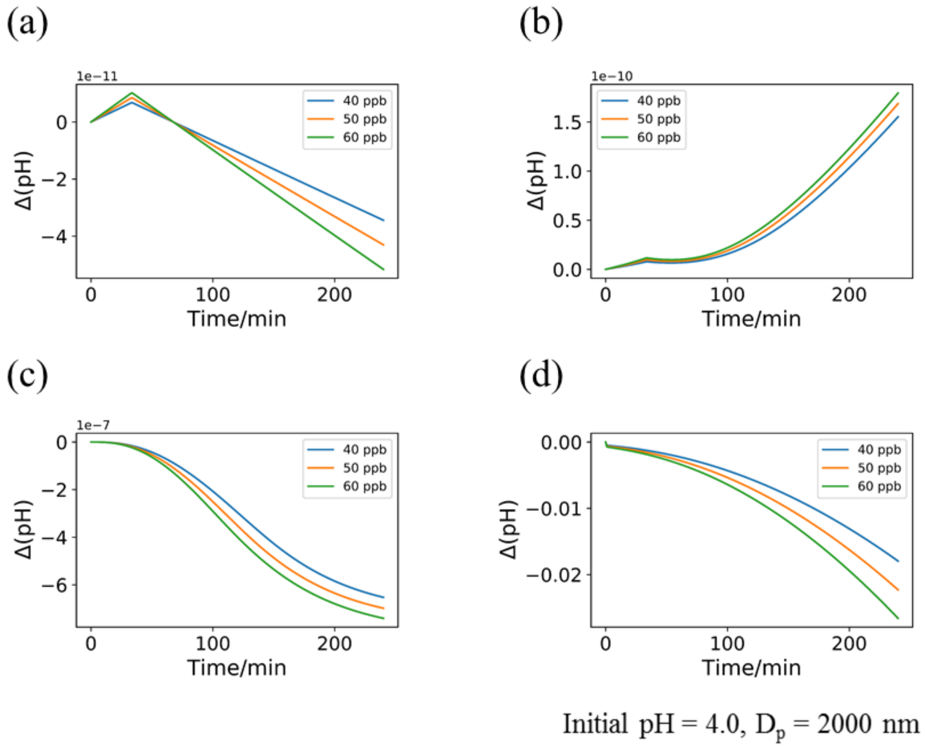

Figure S2. The change of pH value in different SO_2 concentrations for the four scenarios (a-d). The initial pH is 4.0 and the particulate diameter is 2000 nm.

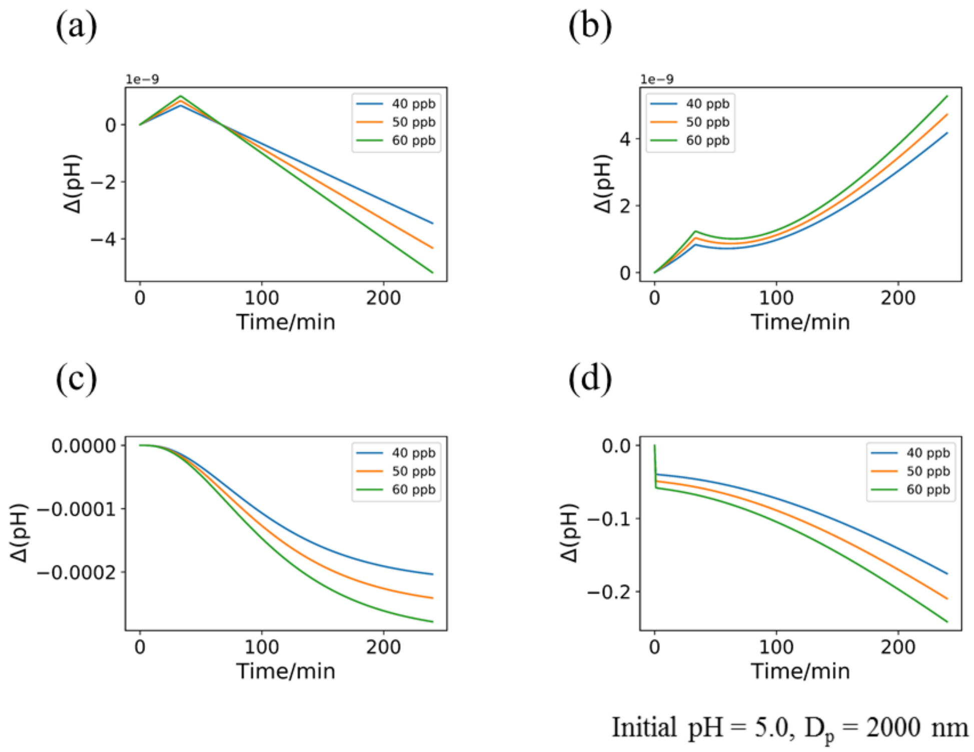

Figure S3. The change of pH value in different SO_2 concentrations for the four scenarios (a-d). The initial pH is 5.0 and the particulate diameter is 2000 nm.

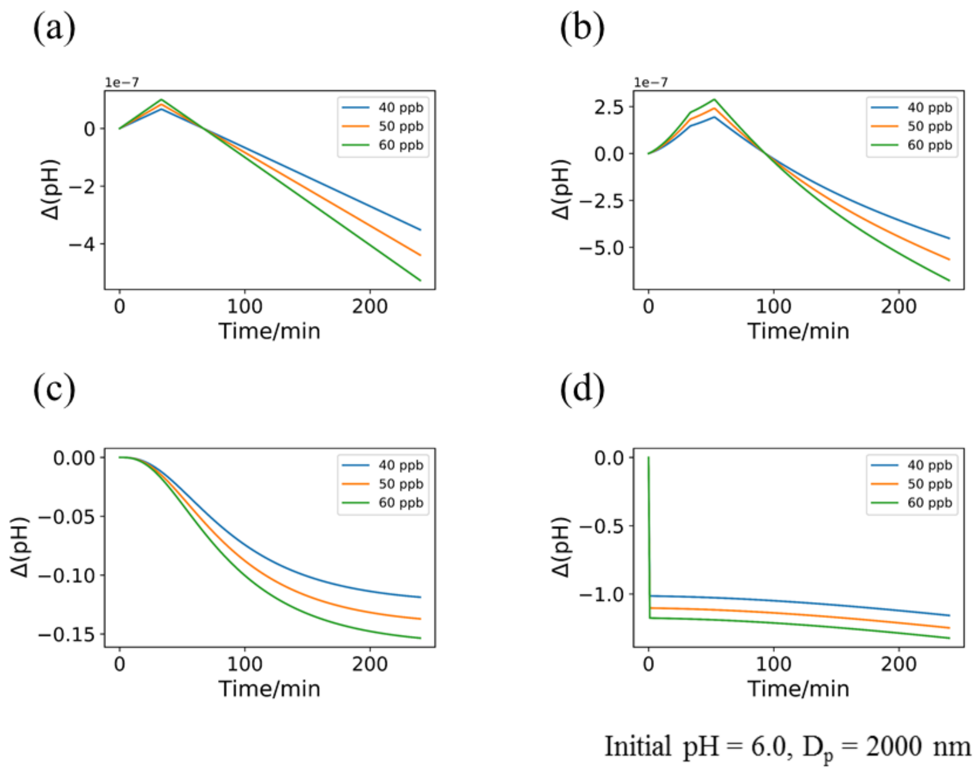

Figure S4. The change of pH value in different SO_2 concentrations for the four scenarios (a-d). The initial pH is 6.0 and the particulate diameter is 2000 nm.

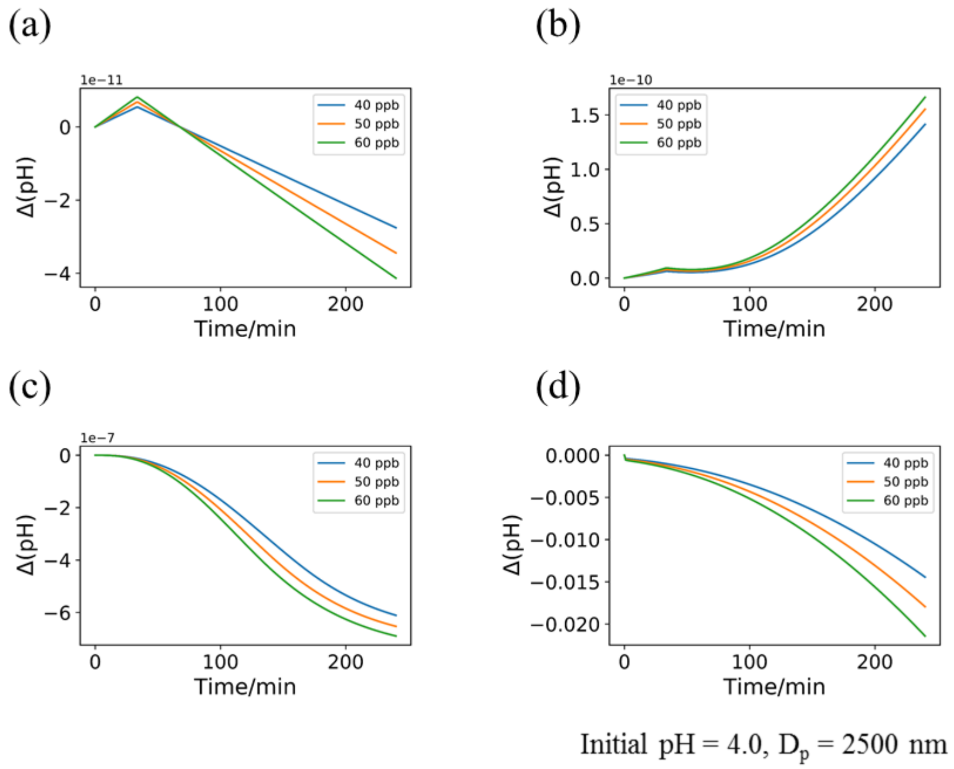

Figure S5. The change of pH value in different SO_2 concentrations for the four scenarios (a-d). The initial pH is 4.0 and the particulate diameter is 2500 nm.

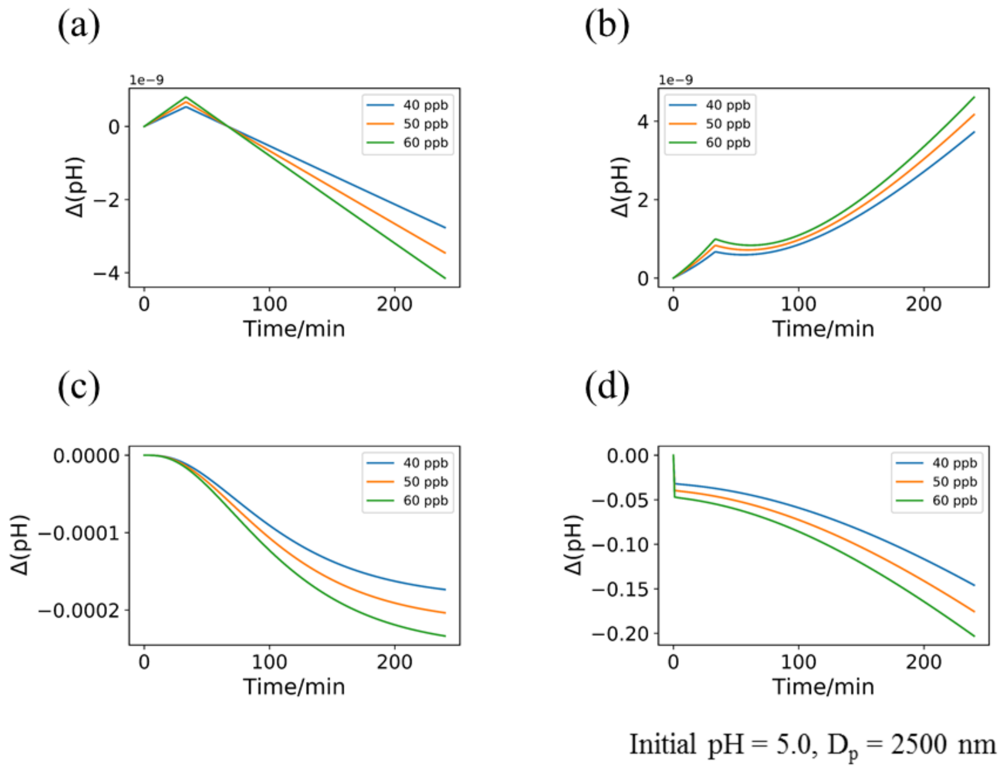

Figure S6. The change of pH value in different SO_2 concentrations for the four scenarios (a-d). The initial pH is 5.0 and the particulate diameter is 2500 nm.

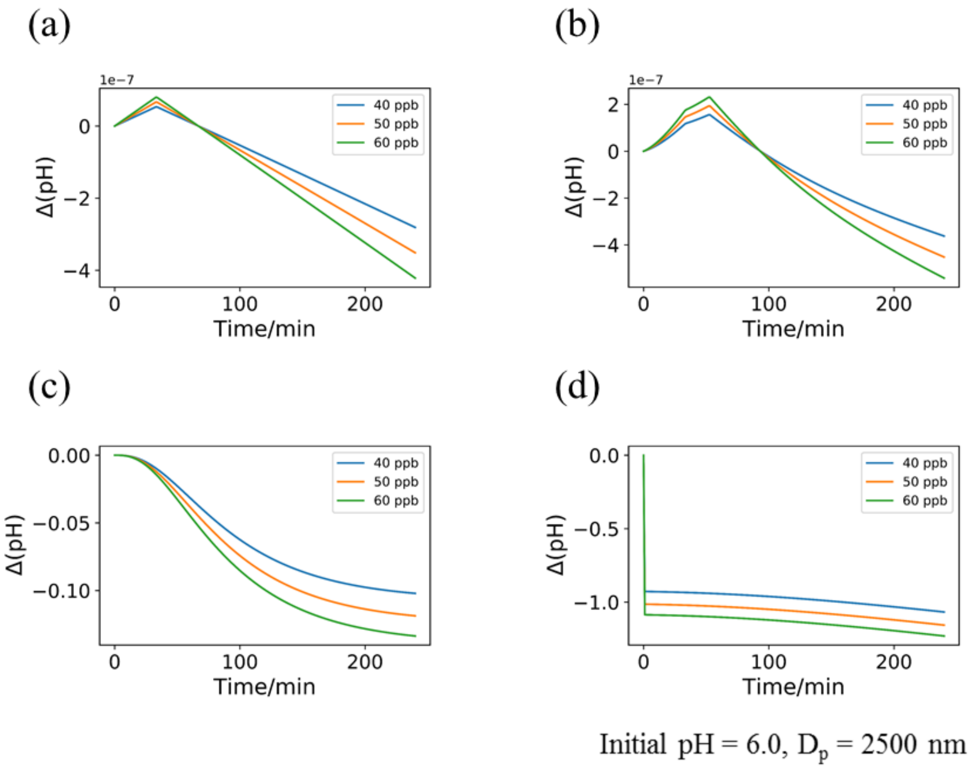

Figure S7. The change of pH value in different SO_2 concentrations for the four scenarios (a-d). The initial pH is 6.0 and the particulate diameter is 2500 nm.

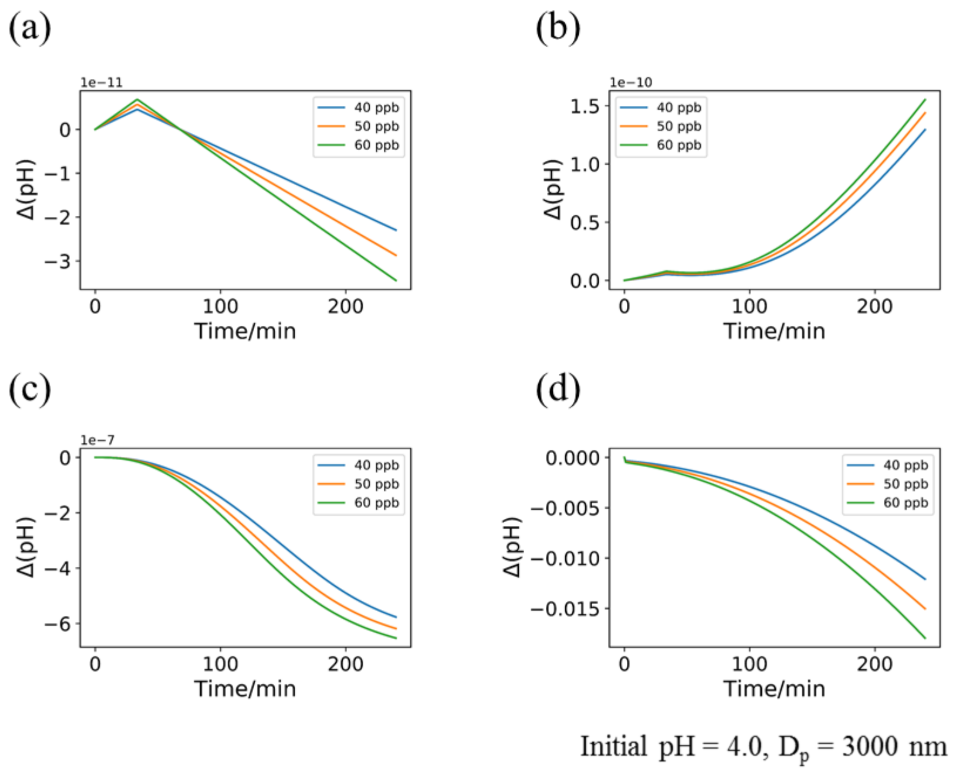

Figure S8. The change of pH value in different SO_2 concentrations for the four scenarios (a-d). The initial pH is 4.0 and the particulate diameter is 3000 nm.

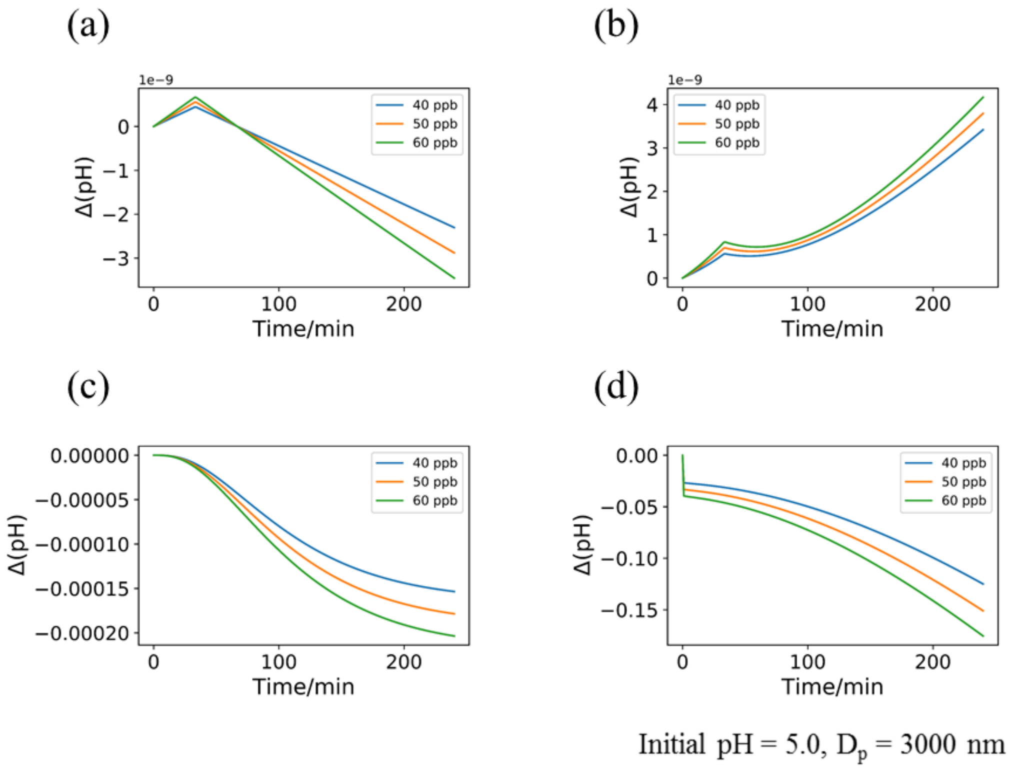

Figure S9. The change of pH value in different SO_2 concentrations for the four scenarios (a-d). The initial pH is 5.0 and the particulate diameter is 3000 nm.

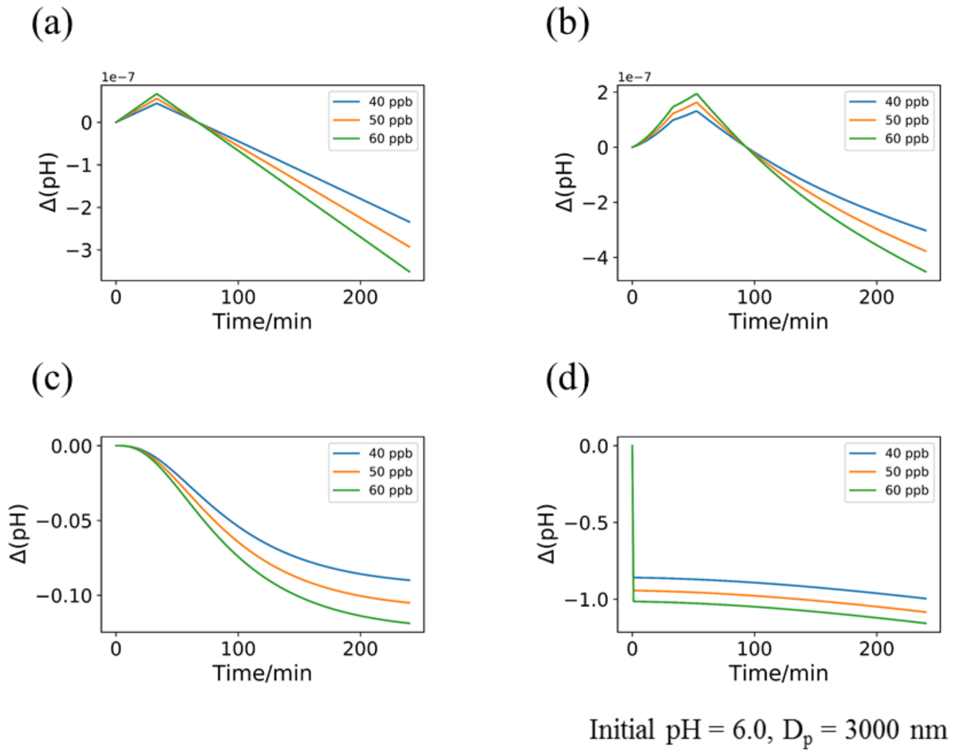

Figure S10. The change of pH value in different SO_2 concentrations for the four scenarios (a-d). The initial pH is 6.0 and the particulate diameter is 3000 nm.

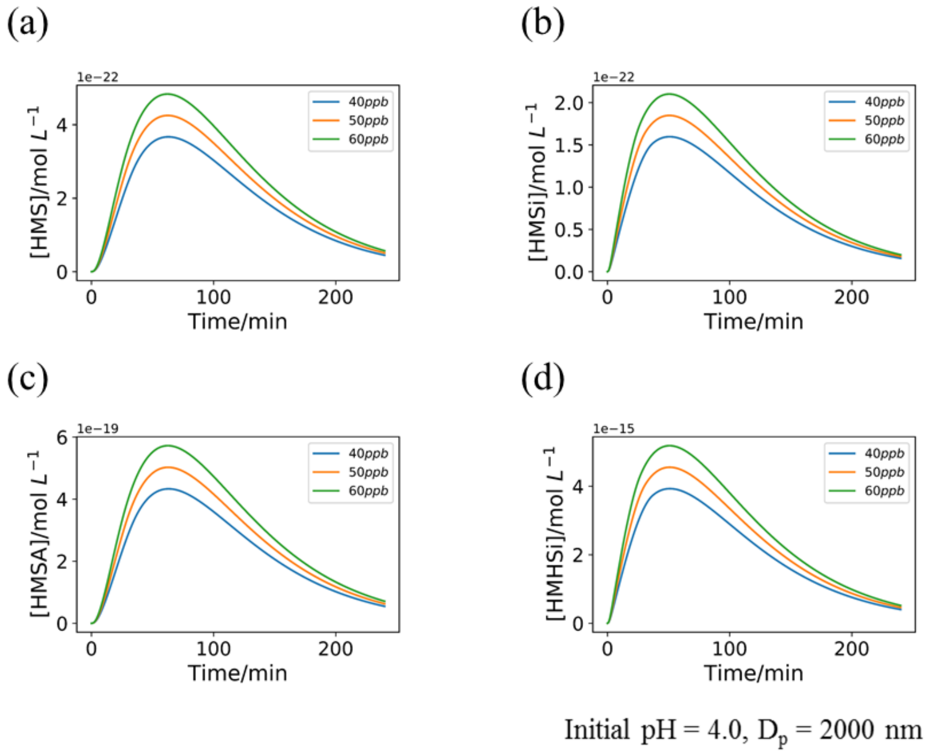

Figure S11. The concentration of (a) HMS, (b) HMSi, (c) HMSA, (d) HMHSi in different SO_2 concentrations. The initial pH is 4.0 and the particulate diameter is 2000 nm.

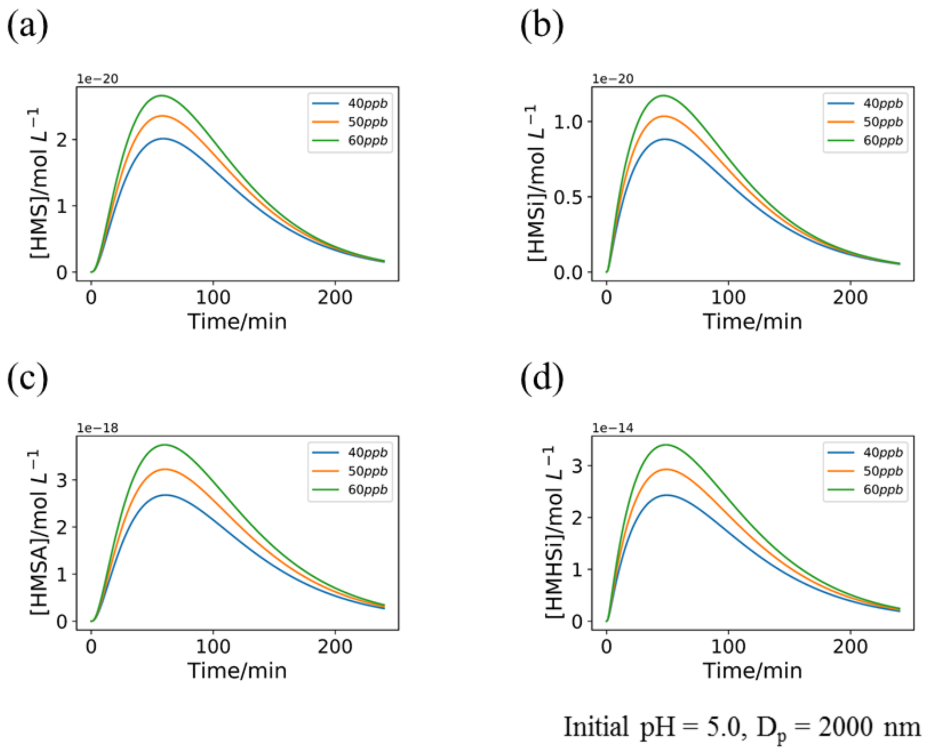

Figure S12. The concentration of (a) HMS, (b) HMSi, (c) HMSA, (d) HMHSi in different SO_2 concentrations. The initial pH is 5.0 and the particulate diameter is 2000 nm.

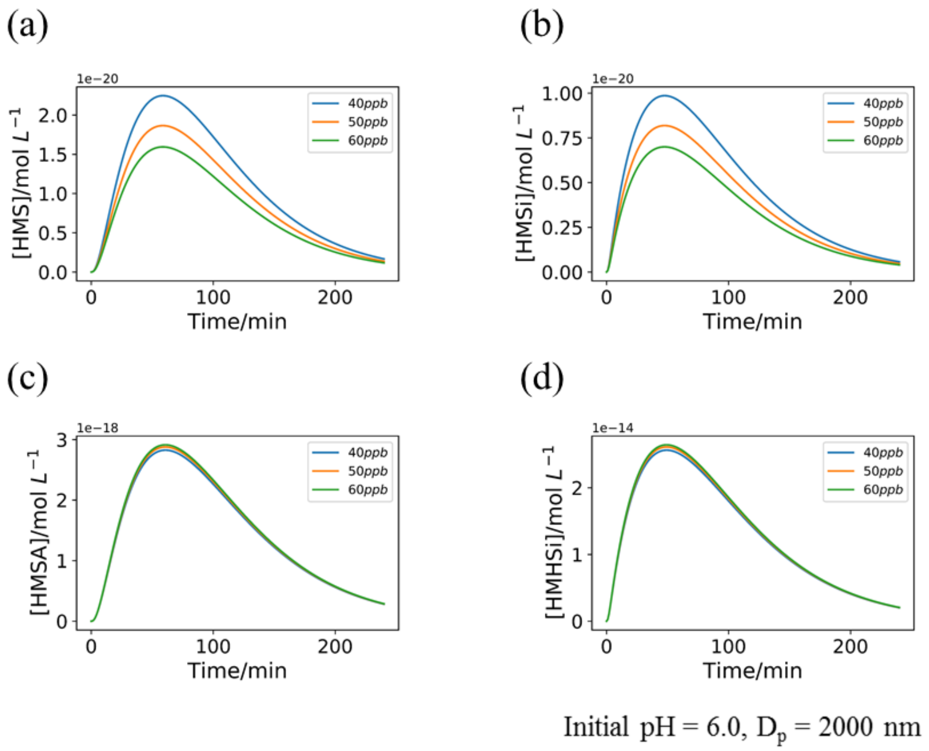

Figure S13. The concentration of (a) HMS, (b) HMSi, (c) HMSA, (d) HMHSi in different SO_2 concentrations. The initial pH is 6.0 and the particulate diameter is 2000 nm.

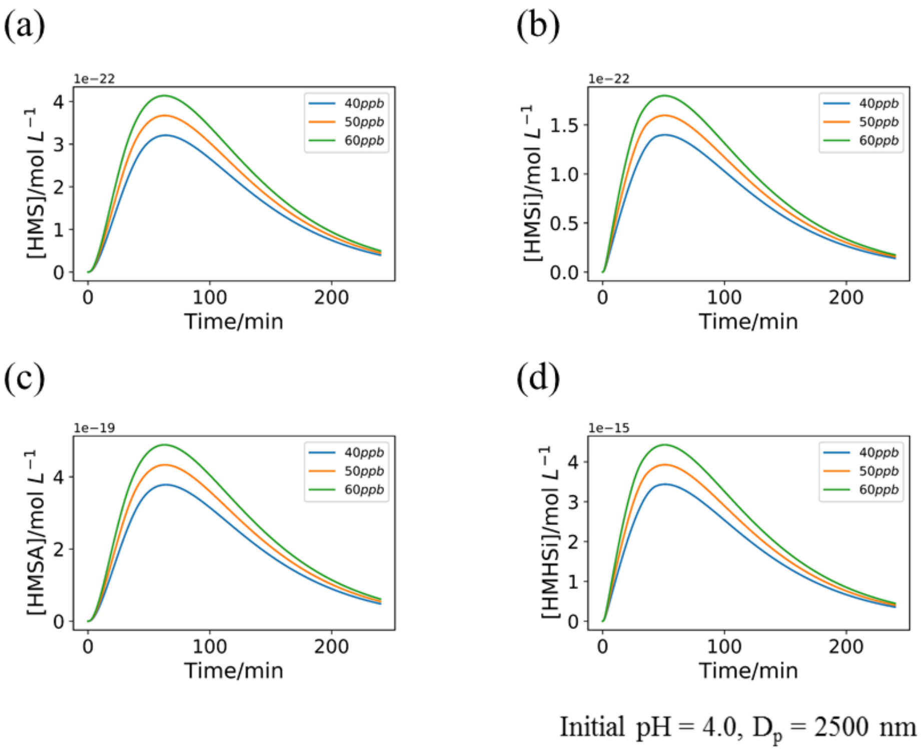

Figure S14. The concentration of (a) HMS, (b) HMSi, (c) HMSA, (d) HMHSi in different SO_2concentrations. The initial pH is 4.0 and the particulate diameter is 2500 nm.

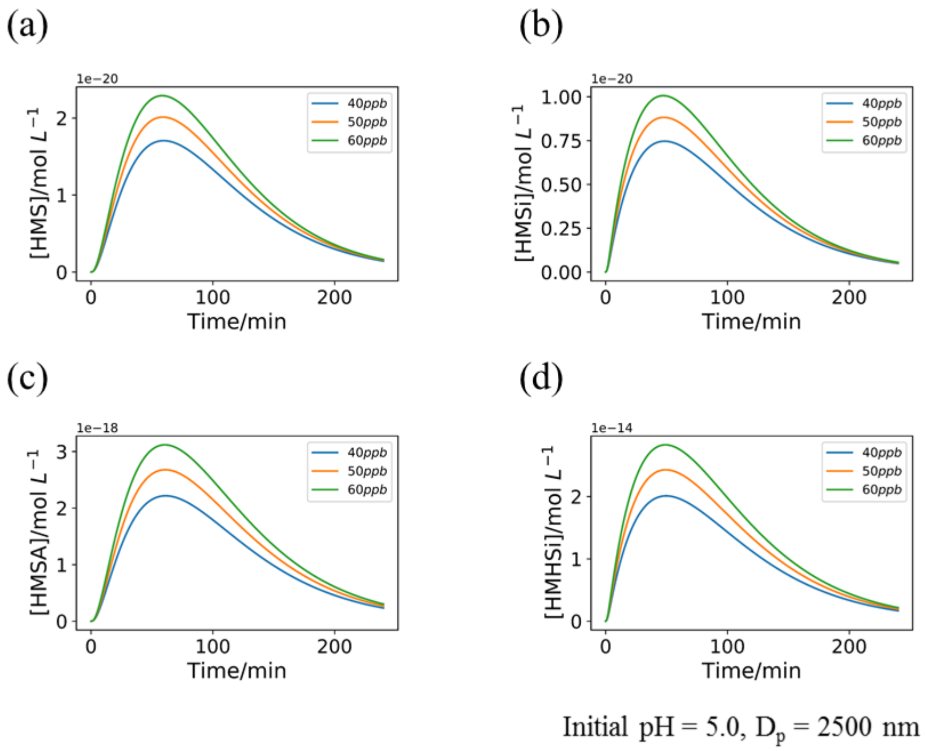

Figure S15. The concentration of (a) HMS, (b) HMSi, (c) HMSA, (d) HMHSi in different SO_2.concentrations. The initial pH is 5.0 and the particulate diameter is 2500 nm.

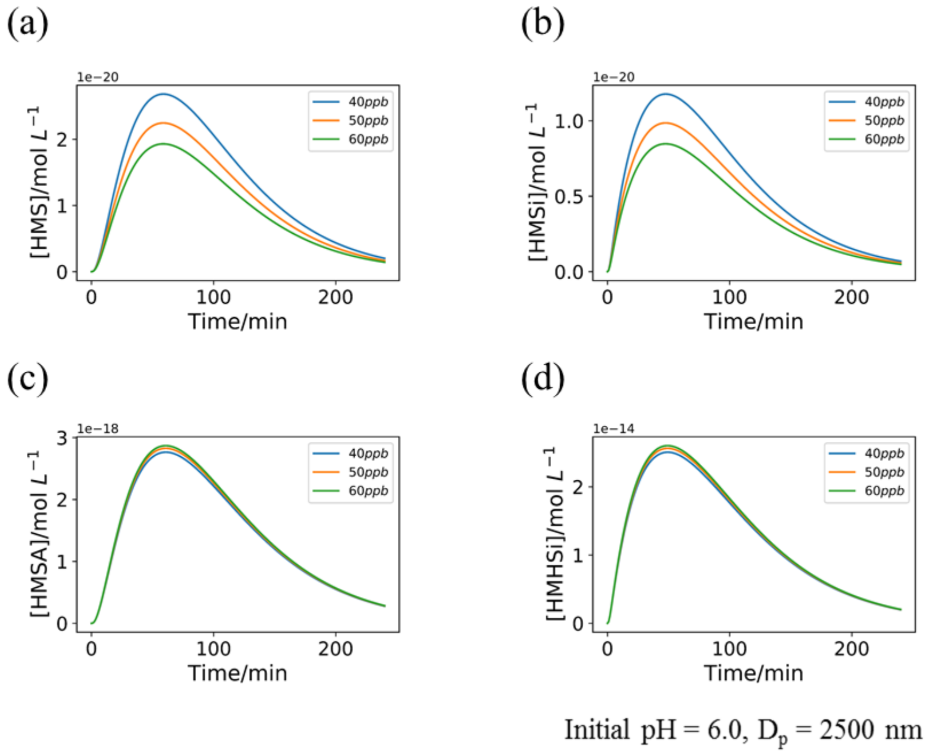

Figure S16. The concentration of (a) HMS, (b) HMSi, (c) HMSA, (d) HMHSi in different SO_2 concentrations. The initial pH is 6.0 and the particulate diameter is 2500 nm.

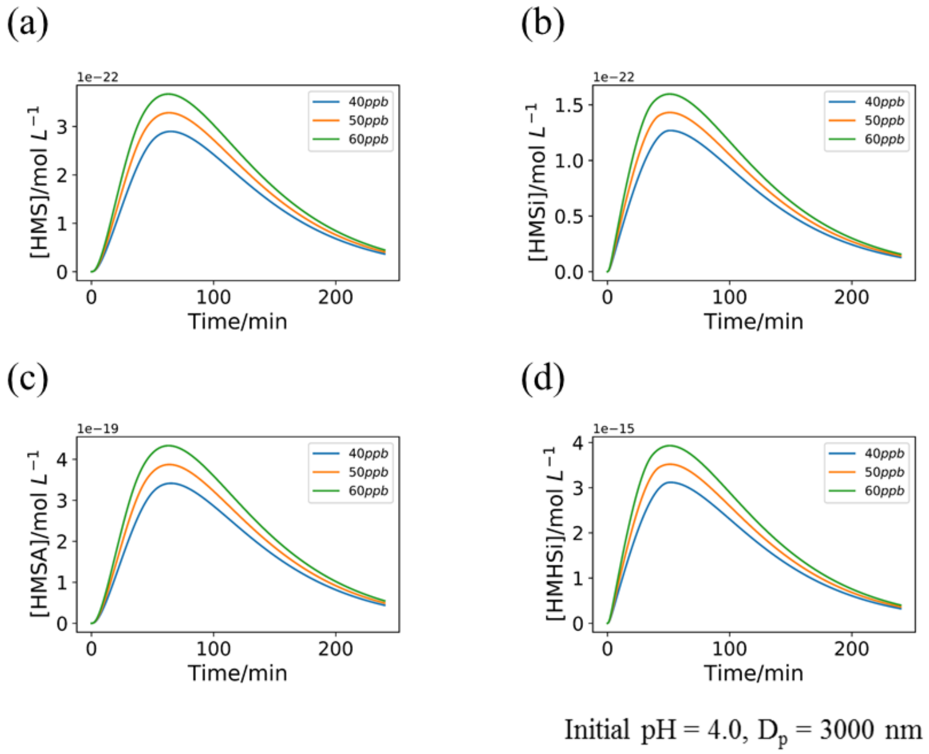

Figure S17. The concentration of (a) HMS, (b) HMSi, (c) HMSA, (d) HMHSi in different SO_2 concentrations. The initial pH is 4.0 and the particulate diameter is 3000 nm.

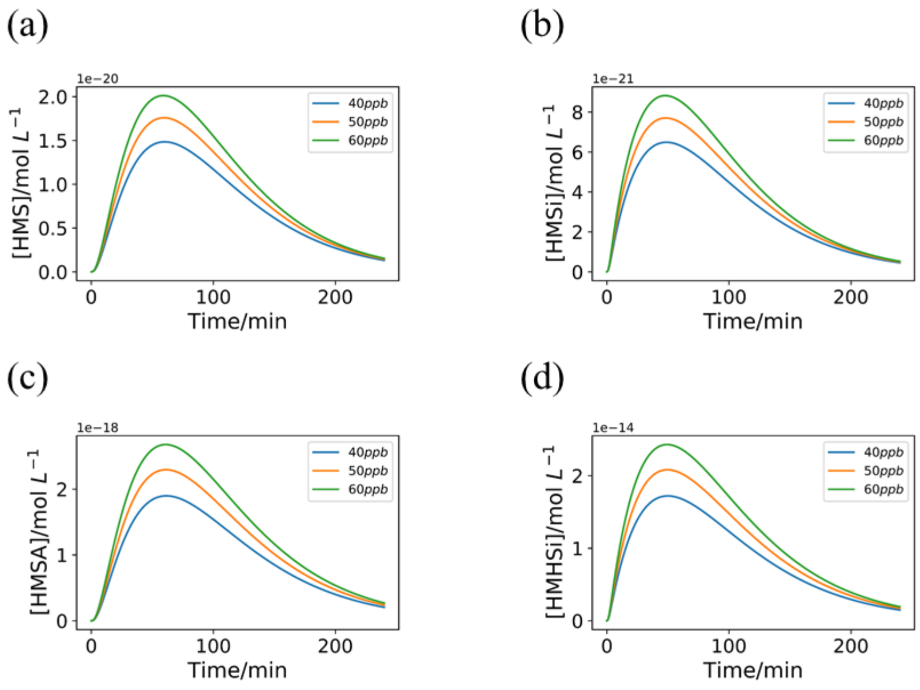

Initial pH = 5.0, D_p = 3000 nm

Figure S18. The concentration of (a) HMS, (b) HMSi, (c) HMSA, (d) HMHSi in different SO_2concentrations. The initial pH is 5.0 and the particulate diameter is 3000 nm.

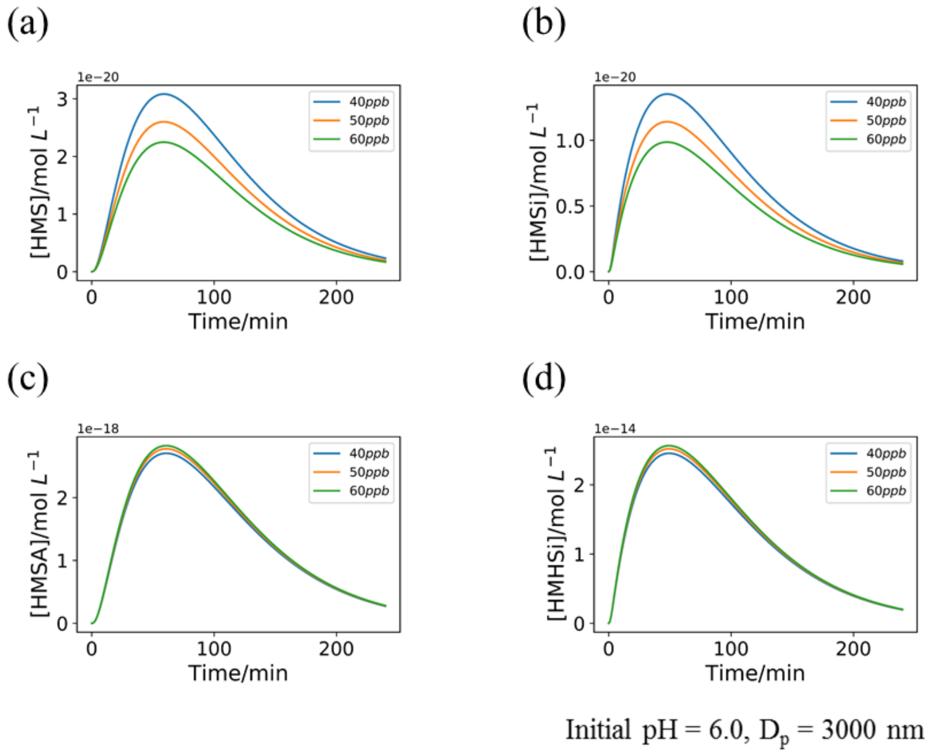

Figure S19. The concentration of (a) HMS, (b) HMSi, (c) HMSA, (d) HMHSi in different SO_2 concentrations The initial pH is 6.0 and the particulate diameter is 3000 nm.

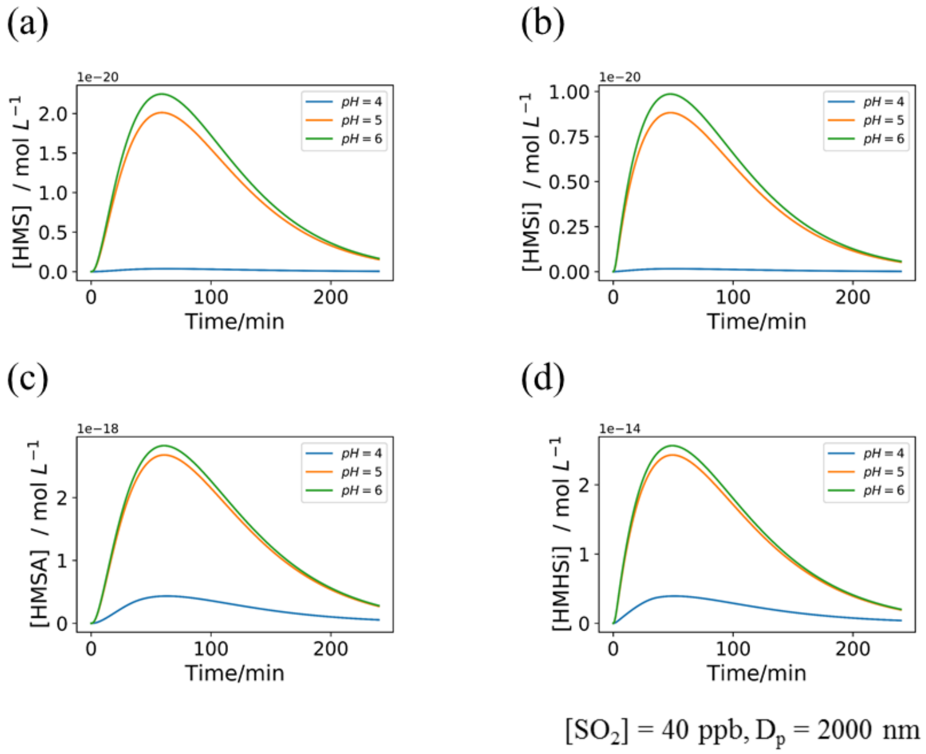

Figure S20. The concentration of (a) HMS, (b) HMSi, (c) HMSA, (d) HMHSi in different initial pH value. The concentration of SO_2 is 40 ppb and the particulate diameter is 2000 nm.

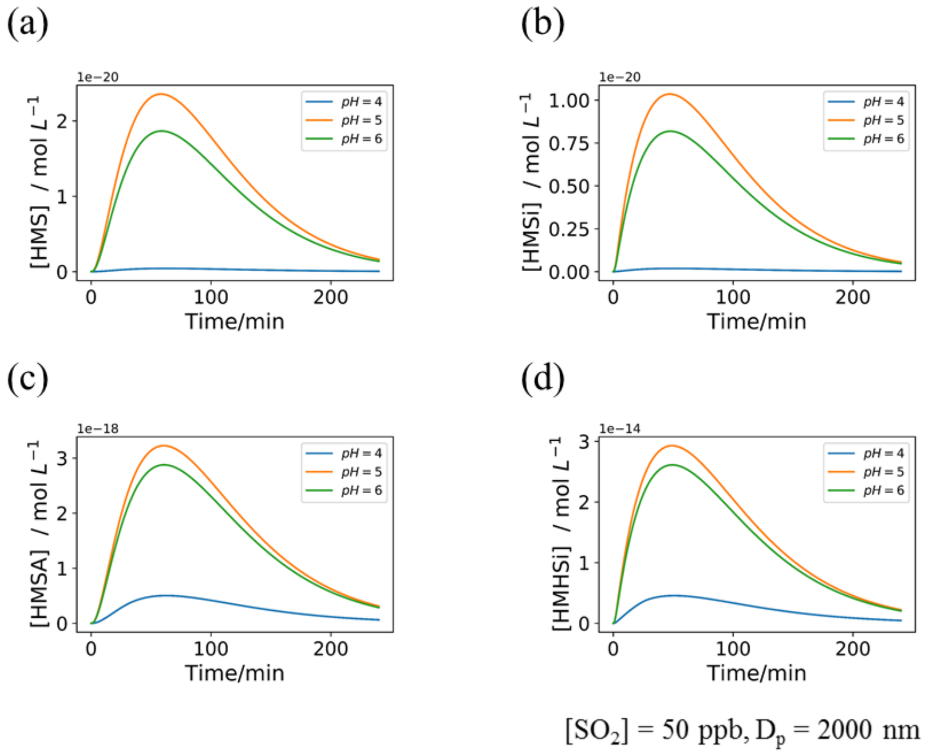

Figure S21. The concentration of (a) HMS, (b) HMSi, (c) HMSA, (d) HMHSi in different initial pH value. The concentration of SO_2 is 50 ppb and the particulate diameter is 2000 nm.

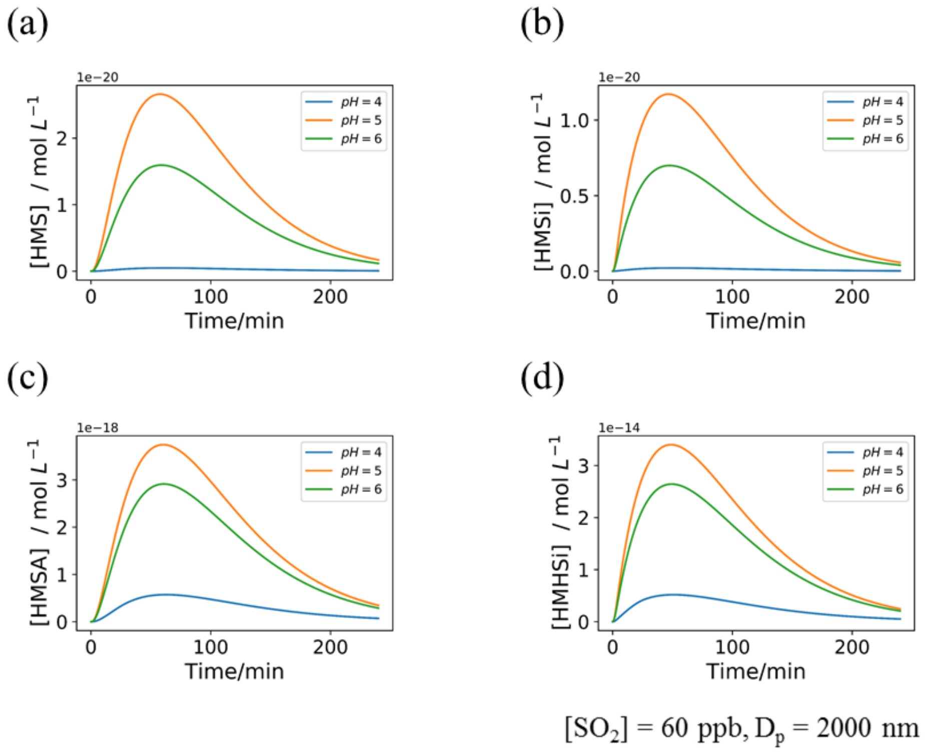

Figure S22. The concentraiton of (a) HMS, (b) HMSi, (c) HMSA, (d) HMHSi in different initial pH value. The concentration of SO_2 is 60 ppb and the particulate diameter is 2000 nm.

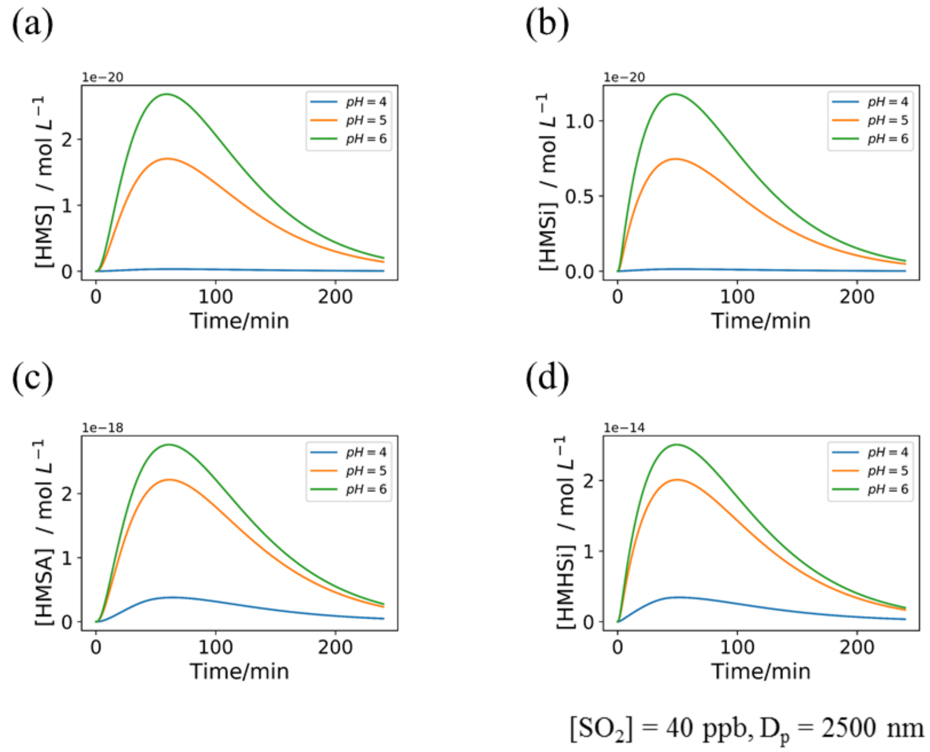

Figure S23. The concentration of (a) HMS, (b) HMSi, (c) HMSA, (d) HMHSi in different initial pH value. The concentration of SO_2 is 40 ppb and the particulate diameter is 2500 nm.

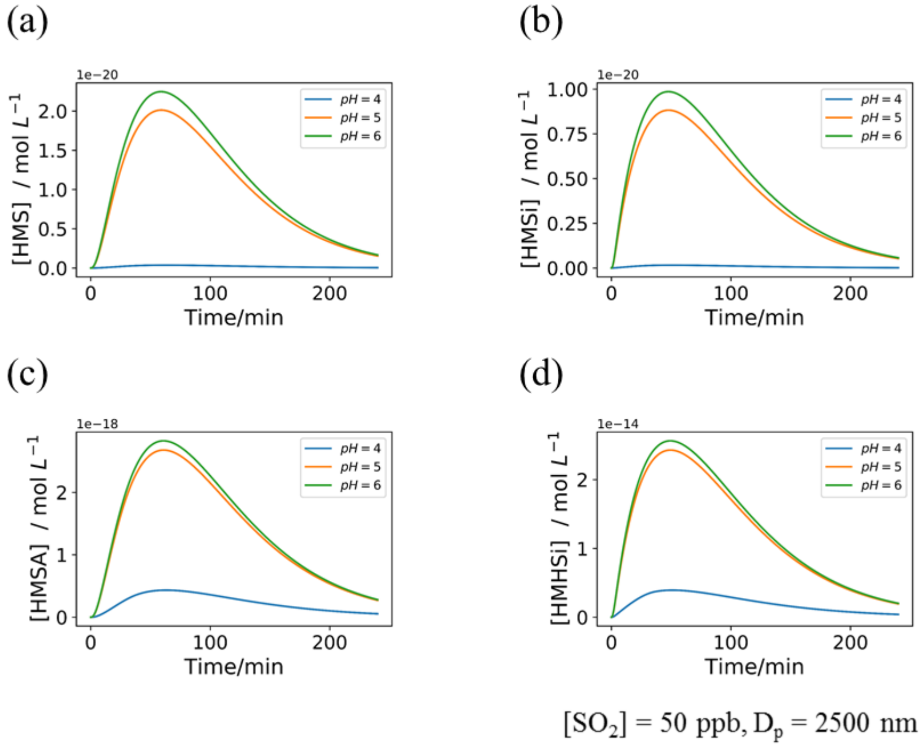

Figure S24. The concentration of (a) HMS, (b) HMSi, (c) HMSA, (d) HMHSi in different initial pH value. The concentration of SO_2 is 50 ppb and the particulate diameter is 2500 nm.

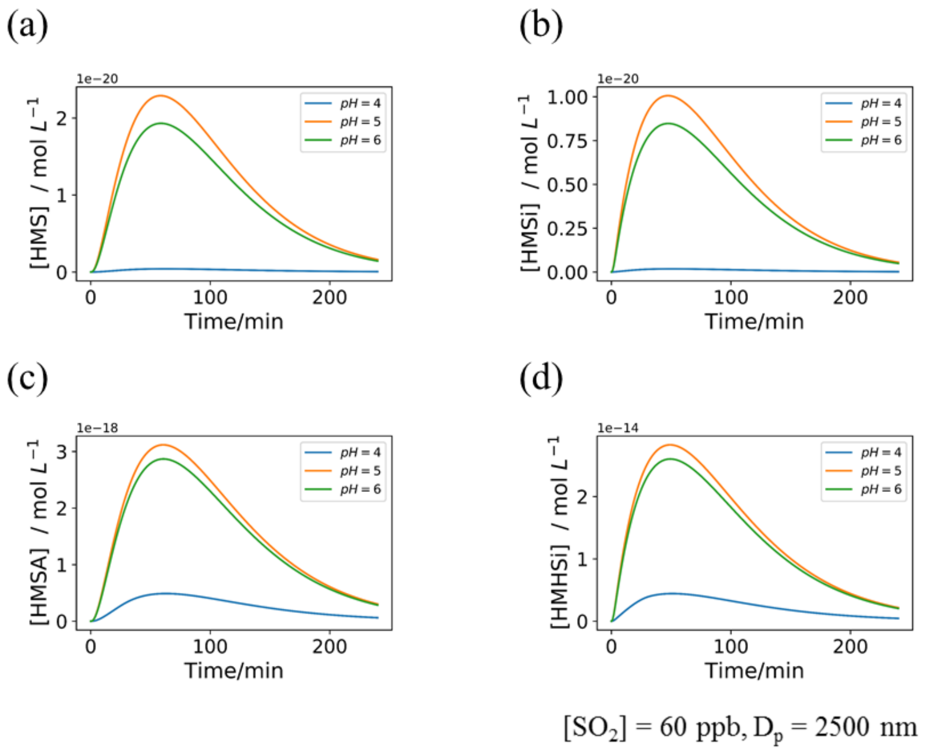

Figure S25. The concentration of (a) HMS, (b) HMSi, (c) HMSA, (d) HMHSi in different initial pH value. The concentration of SO_2 is 60 ppb and the particulate diameter is 2500 nm.

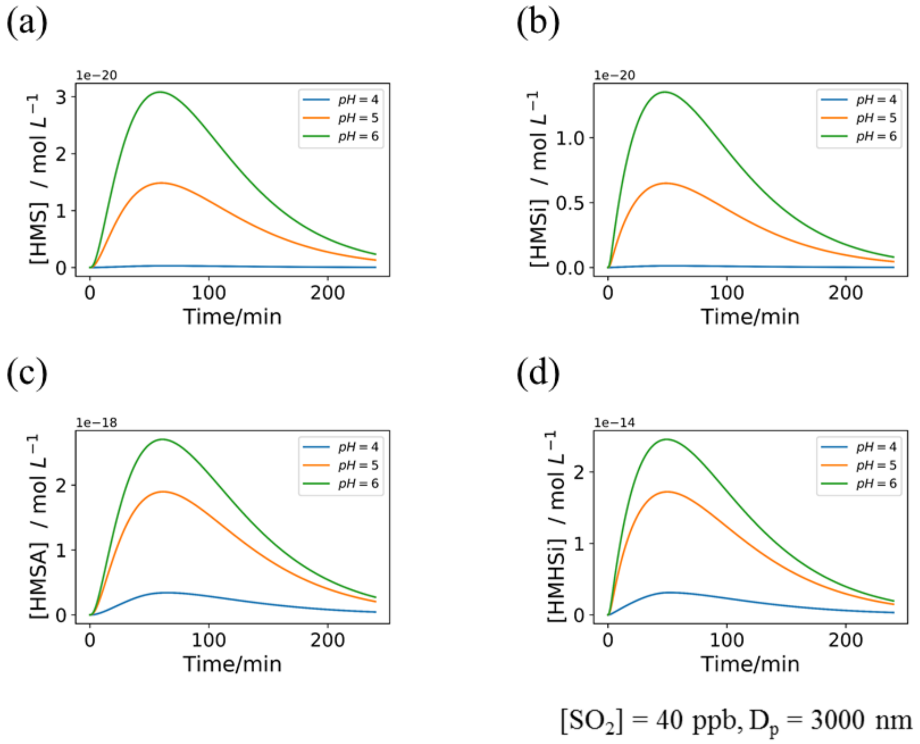

Figure S26. The concentration of (a) HMS, (b) HMSi, (c) HMSA, (d) HMHSi in different initial pH value. The concentration of SO_2 is 40 ppb and the particulate diameter is 3000 nm.

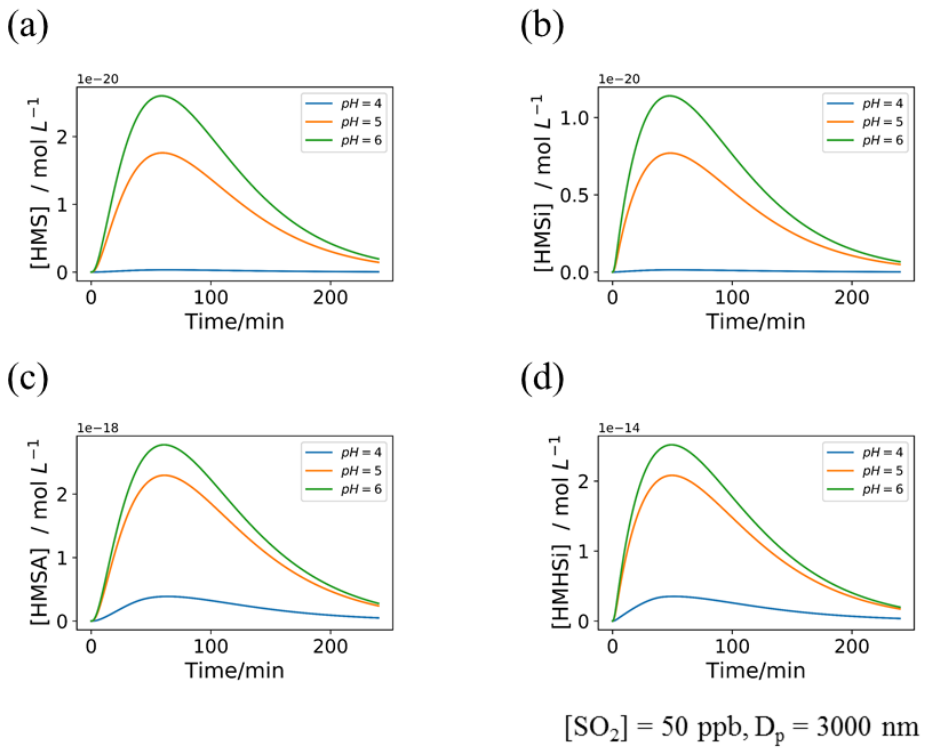

Figure S27. The concentration of (a) HMS, (b) HMSi, (c) HMSA, (d) HMHSi in different initial pH value. The concentration of SO_2 is 50 ppb and the particulate diameter is 3000 nm.

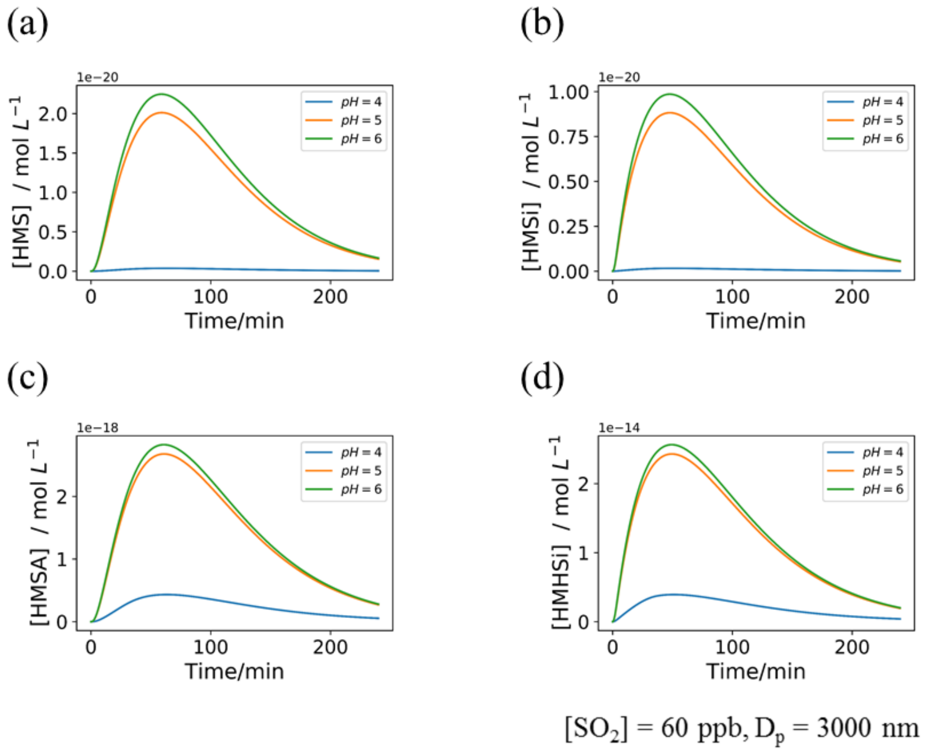

Figure S28. The concentration of (a) HMS, (b) HMSi, (c) HMSA, (d) HMHSi in different initial pH value. The concentration of SO_2 is 60 ppb and the particulate diameter is 3000 nm.

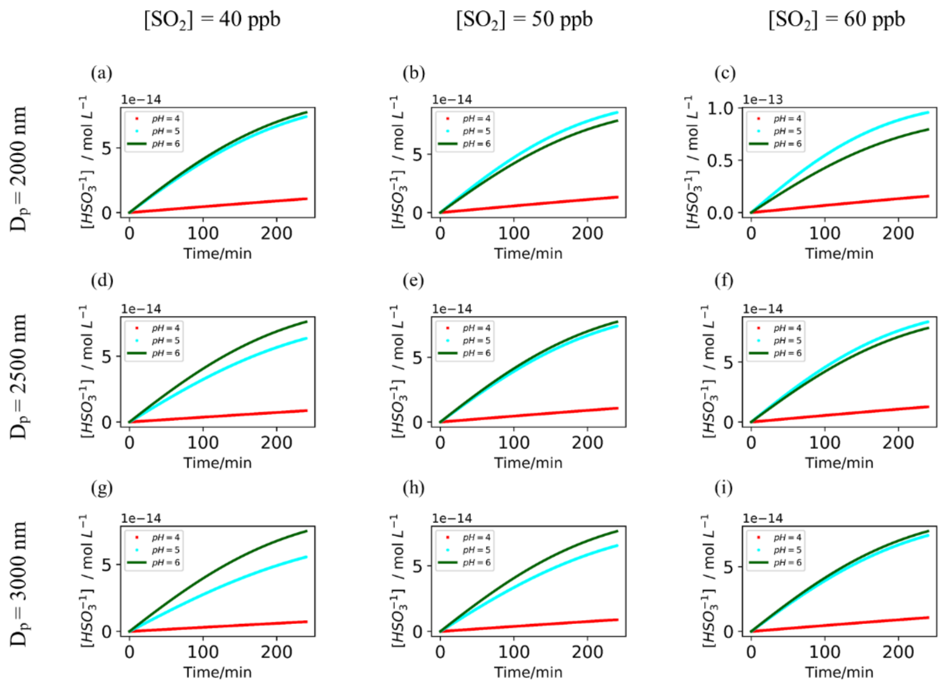

Figure S29. The concentration of HSO_3^- for the fourth scenario with different SO_2 concentration and particulate diameter.

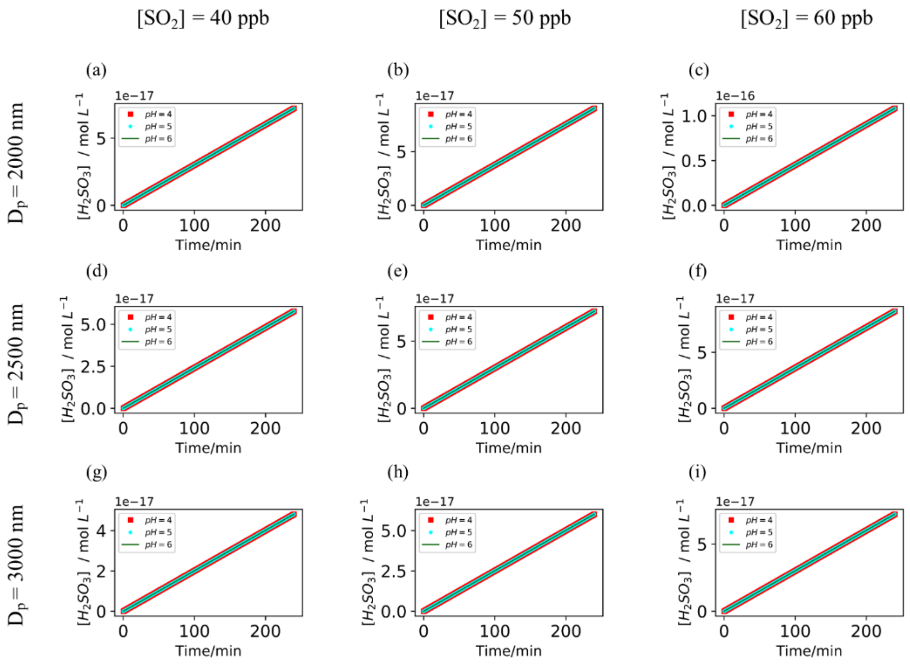

Figure S30. The concentration of H_2SO_3 for the fourth scenario with different SO_2 concentration and particulate diameter.

Text S2

The "in-house" kinetic model is set up for simulations of reactions between bisulfite (also the sulfurous acid) and formaldehyde in atmospheric aqueous aerosols. Our model includes two types of reactions: (1) the uptake of SO_2 into the droplet (SR1), (2) aqueous reactions and equilibria of the compositions (SR2-16), as shown below:

$$SO_2\,(g) + H_2O \rightarrow SO_2{\cdot}H_2O\,(aq) \quad (R1)$$

$$SO_2{\cdot}H_2O \rightleftharpoons H_2SO_3 \quad (R2)$$

$$H_2SO_3 + H_2O \rightleftharpoons HSO_3^- + H_3O^+ \quad (R3)$$

$$HSO_3^- + HCHO \rightleftharpoons CH_2(OH)SO_3^-(HMS) \quad (R4)$$

$$HSO_3^- + HCHO \rightleftharpoons CH_2(OH)OSO_2^-(HMSi) \quad (R5)$$

$$HSO_3^- + HCHO + H_2O \rightleftharpoons CH_2(OH)SO_3^-(HMS) + H_2O \quad (R6)$$

$$HSO_3^- + HCHO + H_2O \rightleftharpoons CH_2(OH)OSO_2^-(HMSi) + H_2O \quad (R7)$$

$$HSO_3^- + HCHO \rightleftharpoons CH_2(OH)SO_3^-(HMS) \quad (R8)$$

$$HSO_3^- + HCHO \rightleftharpoons CH_2(OH)OSO_2^-(HMSi) \quad (R9)$$

$$H_2SO_3 + HCHO \rightleftharpoons CH_2(OH)SO_3H\ (HMSA) \quad (R10)$$

$$H_2SO_3 + HCHO \rightleftharpoons CH_2(OH)OSO_2H\ (HMHSi) \quad (R11)$$

$$H_2SO_3 + HCHO + H_2O \rightleftharpoons CH_2(OH)SO_3H\ (HMSA) + H_2O \quad (R12)$$

$$H_2SO_3 + HCHO + H_2O \rightleftharpoons CH_2(OH)OSO_2H\ (HMHSi) + H_2O \quad (R13)$$

$$HCHO + H_2O \rightleftharpoons CH_2(OH)_2 \quad (R14)$$

$$CH_2(OH)SO_3H\ (HMSA) + H_2O \rightleftharpoons CH_2(OH)SO_3^-\ (HMS) + H_3O^+ \quad (R15)$$

$$CH_2(OH)OSO_2H\ (HMHSi) + H_2O \rightleftharpoons CH_2(OH)OSO_2^-\ (HMSi) + H_3O^+ \quad (R16)$$

The uptake rate of sulfur dioxide onto the droplet is evaluated as

$$R_{SO_2}^{uptake} = \frac{1}{4}\gamma[SO_2]v_{smr}A_s\,. \qquad \text{(Equ. 1)}$$

Here γ is the uptake coefficient of SO_2 on a water droplet, v_{smr} is the square-mean-root velocity of SO_2, $[SO_2]$ is the concentration of SO_2 in the air, A_s is the surface area of the droplet. The reaction rates of SR2-16 are evaluated by the law of mass action. Taking SR4 as an example, the rate of the forward reaction is evaluated as

$$R_{forward} = k_{forward,4}[HCHO][HSO_3^-]\,. \qquad \text{(Equ. 2)}$$

Here $k_{forward,4}$ is the reaction rate constant of the forward reaction, [HCHO] and $[HSO_3^-]$ are the concentrations of HCHO and HSO_3^- in the simulating droplet, respectively.

During the simulations, the steepest descent method is used to solve the nonlinear equations with the convergence limit as 1.0×10^{-3}. Data is recorded after every unit simulating timestep (one second), and the simulating results can be calibrated every sixty simulating timesteps (one minute) by comparing with the equilibrium constants. Taking SR15 as an example, the standard chemical equilibrium constant ($K_{HMS/HMSA}$) is formulated as

$$K_{HMS/HMSA} = \frac{([HMS]/c_0)([H_3O^+]/c_0)}{([HMSA]/c_0)}\,, \qquad \text{(Equ. 3)}$$

where [HMS], $[H_3O^+]$ and [HMSA] is the concentration of HMS, H_3O^+ and HMSA, respectively. Since the denominator, $[HMSA]/c_0$, is very small which can induce huge numerical error, Equ.3 is transformed into

$$K_{HMS/HMSA}([HMSA]/c_0) - ([HMS]/c_0)([H_3O^+]/c_0) = 0\,, \qquad \text{(Equ.4)}$$

and hence the relative bias (Δ^{re}) is defined as calibration

$$\Delta^{re} = \frac{\left|K_{HMS/HMSA}([HMSA]/c_0) - ([HMS]/c_0)([H_3O^+]/c_0)\right|}{K_{HMS/HMSA}([HMSA]/c_0)}\,. \qquad \text{(Equ.5)}$$

The allowed maximum value of Δ^{re} is set as 1.0×10^{-3}, and if Δ^{re} is larger than this value,

calibration will be carried out as

$$[\mathrm{HMSA}] = \frac{([\mathrm{HMS}]/c_0)([\mathrm{H_3O^+}]/c_0)}{K_{\mathrm{HMS/HMSA}}/c_0}. \qquad \text{(Equ.6)}$$

As for other equilibria, the formulations of Δ^{re} and calibration equations are defined in a similar way. Note that the concentrations of HMS and HMSA (also HMSi and HMHSi) are tremendously lower than those of other species. However, they are the most important in our study, Hence, the other form of calibration is introduced,

$$[\mathrm{HMS}] = \frac{K_{\mathrm{HMS/HMSA}}[\mathrm{HMSA}]}{([\mathrm{H_3O^+}]/c_0)}, \qquad \text{(Equ.7)}$$

and in practice, to avoid [HMS] or [HMSA] being deviated by the accumulating error, Equ.6 is used in odd-number calibrations and Equ.7 is used in even-number calibrations. The same treatment can be employed for SR16 to calibrate [HMSi] and [HMHSi].
